# A scalable Tn5-based method for genome-wide DNA methylation profiling in development and disease

**DOI:** 10.1038/s41467-026-73325-4

**Published:** 2026-05-22

**Authors:** Hanrong Hu, Nahuel Simonet, Ece Naz Bilgiç, Heather Murray, Regina Reimann, Markus Rechsteiner, Fides Zenk

**Affiliations:** 1 Ecole Polytechnique Federale de Lausanne (EPFL), School of Life Sciences, Brain Mind Institute, EpiGN—NeuroNA Chair in Epigenomics of Neurodevelopmental Disorders, Station 19, Lausanne, Switzerland; 2 Campus Biotech, Chemin des Mines 9, Geneve, Switzerland; 3 UniversitätsSpital Zürich, Schmelzbergstrasse 12, Zürich, Switzerland

**Keywords:** Epigenomics, Epigenomics, Methylation analysis

## Abstract

DNA methylation is a key epigenetic modification involved in development and disease, including cancer, and serves as a biomarker for diagnosis. Current detection methods, such as bisulfite sequencing, provide base-pair resolution but require high sequencing depth and cost. Here, we developed C^me^CUT&Tag, a Tn5-based approach that uses methylation-binding domain fusion proteins to selectively target methylated DNA in chromatinized and isolated DNA. This enables adapter insertion into methylated regions, allowing low-depth sequencing for quantitative analysis or optional cytosine conversion for base-pair resolution. C^me^CUT&Tag enables genome-wide DNA methylation profiling with reduced input and sequencing requirements. We demonstrate its performance in characterizing DNA methylation across development and disease in human stem cells, organoids, zebrafish embryogenesis, and tumor biopsies. The method shows strong concordance with bisulfite sequencing and supports the classification of brain tumor samples into methylation subtypes. These features make C^me^CUT&Tag a scalable and cost-effective approach for epigenetic research and potential clinical applications.

## Abstract (web summary)

DNA methylation is a key epigenetic mark in development and disease. Here, the authors present CmeCUT&Tag, a cost-effective method that profiles methylation in cells, tissues, and tumors with low input, enabling high-throughput and single-cell applications.

## Introduction

DNA methylation detection faces a fundamental trade-off between resolution, cost, and throughput that has limited its widespread adoption in biomedical research. While DNA methylation is a crucial epigenetic modification implicated in many diseases, including cancer^1^, and has been used as a biomarker for early disease detection, aging, and tumor classification^2,3^, current detection methods impose constraints on experimental design and accessibility. Whole Genome Bisulfite Sequencing (WGBS), the current gold standard, converts unmethylated cytosines to uracil (read as thymine during sequencing) while preserving methylated cytosines^4^. This approach provides base-pair resolution but demands extraordinary sequencing depth; current recommendations require 30× coverage per locus, translating to 800 million to 1 billion read pairs for human genome coverage. Alternative enrichment strategies either use methylation-sensitive restriction enzymes^5^ (RRBS), microarrays (Infinium MethylationEPIC array) or antibodies against 5-methylcytosine^6^ or methylation-binding domains^7,8^, which offer cost reduction but either lack base-pair resolution, require substantial input material (between 1–5 µg of chromatin or purified DNA^6–8^), suffer from variable specificity, or cannot perform in situ in nuclei, which makes them unsuitable for biomedical applications and droplet-based single-nucleus sequencing technologies^6^.

CUT&Tag has become the go-to high-throughput, low-input, cost-efficient method for mapping a variety of epigenetic modifications and transcription factors^9–11^. Nevertheless, in its current setup, it fails to enrich for DNA methylation via antibody-mediated Tn5 binding. In this work, to map enrichment of DNA-methylation cost-effectively and in high-throughput, we have developed C^me^CUT&Tag, a strategy to enrich regions of high DNA methylation using fusion proteins of different DNA methylation binding domains (MBD, e.g., of MeCP2, MBD2) and a hyperactive Tn5.

Our study presents a Tn5-based approach for high-throughput DNA methylation profiling that enables mapping of methylation patterns both in situ in nuclei and on isolated DNA. By combining methylation-binding domains with Tn5, the method allows efficient enrichment of methylated regions with low input and reduced sequencing depth requirements. This enables scalable and cost-effective analysis across diverse biological systems, including stem cells and organoids, where we capture dynamic changes in DNA methylation during development. The approach is modular and compatible with single-cell and cytosine-conversion-based workflows, providing a flexible framework for studying epigenetic regulation in both basic and translational contexts.

## Results

### MBD–Tn5 fusion design for DNA methylation targeting

**Fig. 1:**
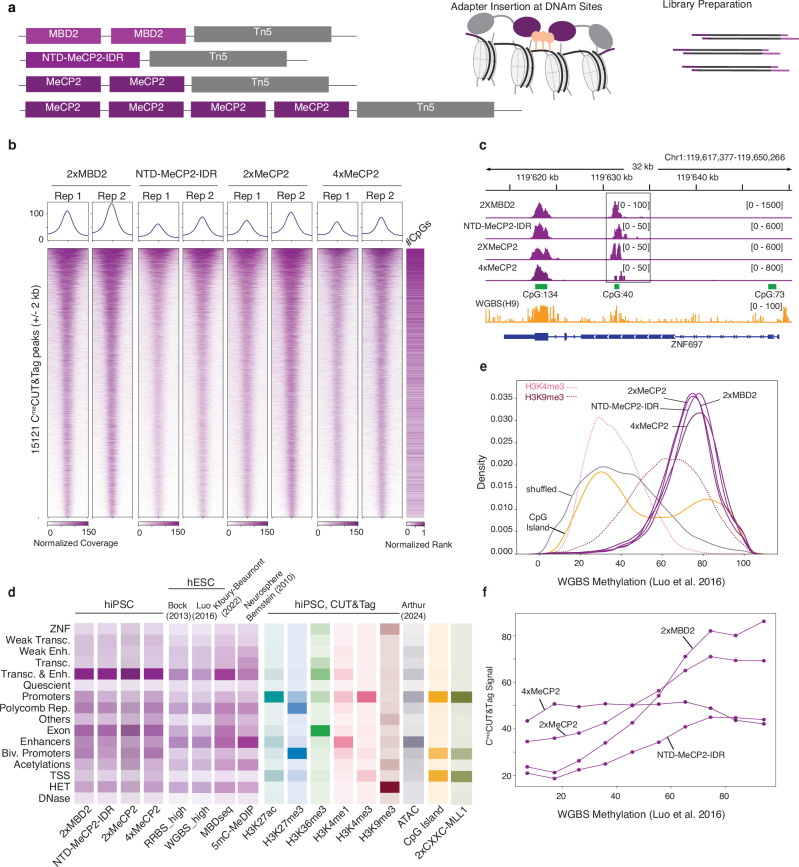
Characterization of MBD-coupled Tn5. **a** Schematic of the MBD-Tn5 fusion proteins and the principle of targeting methylated DNA regions in the genome. The DNA methylation-binding domains (MBDs) of MBD2 and MeCP2 were fused to Tn5. NTD-MeCP2-IDR also contains the N-terminal domain and the intrinsically disordered region adjacent to the MBD. **b** Heatmaps showing binding profiles of different MBD constructs in iPSCs over the consensus of enriched regions (peaks), sorted by signal intensity. All constructs show similar enrichment patterns (*n* = 2 per construct, see Supplementary Fig. 2a for correlation analysis). The adjacent CpG density profile (rank-normalized) demonstrates preferential MBD binding at CpG-rich loci. **c** Examples of MBD binding to CpG-rich genomic regions at the *ZNF697* locus, along with whole-genome bisulfite sequencing (WGBS) data from human embryonic stem cells (H9)^18^. MBD constructs effectively capture methylated CpG islands. **d** Chromatin state annotation from ChromHMM shows that MBD binding is enriched at specific chromatin states and coincides with regions of high DNA methylation ( > 75% average mCpG) in published WGBS^18^ and RRBS^5^ (reduced representation bisulfite sequencing) datasets on human embryonic stem cells, and previously published enrichment-based methods (MeDIP on cortex-derived neurospheres^20^ and MBDseq on hESCs^19^). The enrichment profiles of MBD-Tn5 are distinct from classic CUT&Tag enrichments for histone modifications, ATAC, and a Tn5 coupled to the CXXC domain of MLL1. **e** Density plot showing the distribution of average DNA methylation levels (% mCpG, from WGBS in H9 cells^18^) across peaks identified by C^me^CUT&Tag and histone modifications measured by CUT&Tag. C^me^CUT&Tag peaks are predominantly located in regions with >40% DNA methylation, highlighting its specificity for highly methylated loci. Shuffled represents the distribution of average DNA methylation levels calculated from a random set of genomic regions matched for region length distribution. **f** Correlation of C^me^CUT&Tag (BigWig) signal with DNA-methylation signal measured by WGBS^18^ of all Tn5 fusion proteins on the C^me^CUT&Tag peaks.

We constructed fusion proteins by individually and combinatorially fusing the DNA methylation binding domains of MBD2 and MeCP2 to Tn5 (Fig. 1a)^12–14^. The resulting recombinant proteins, 2xMBD2-Tn5, NTD-MeCP2-IDR-Tn5 (NTD-N-terminal domain, IDR-intrinsically disordered region), 2xMeCP2-Tn5, and 4xMeCP2-Tn5, were expressed in *Escherichia coli* and subsequently purified using a chitin resin to ensure high purity (Supplementary Fig. 1a, b). We selected these domains based on their successful application in MethylCap-Seq^7^ and MiGS^8^ and aimed to optimize DNA methylation-binding efficiency by drawing on prior studies. Reports have shown that MeCP2 binding is enhanced by incorporating four tandem domains^12^, as well as the addition of its intrinsically disordered region (IDR) and N-terminal domain (NTD)^15^. Moreover, mouse MBD2 has been demonstrated to possess the highest affinity for methylated DNA, as measured by CEMSA^14^. While the genome-wide binding patterns of full-length MBD proteins, MBD1, MBD2, MBD4, and MeCP2, are broadly similar in mouse embryonic stem cells, they also exhibit some protein-specific interactions^16^. In contrast, MBD3 does not appear to enrich at methylated DNA loci^17^. Nonetheless, the MBD domains of these proteins (with the exception of MBD3) are highly conserved, and the differences in genome-wide binding likely reflect variations in protein domain and complex composition rather than intrinsic DNA-binding specificity^16^.

### MBD–Tn5 enriches CpG-rich methylated regions in iPSCs

Initial characterization in human induced pluripotent stem cells (iPSCs) (Supplementary Fig. 1c–f) revealed strong enrichment in CpG-rich regions across all variants (Fig. 1b). The individual domain combinations show remarkably similar enrichment profiles, with Pearson correlations >0.8 between biological replicates and different constructs, and a comparable fraction of reads in peaks (FRiP scores >0.35) (Fig. 1b, c, and Supplementary Fig. 2a–e), and outperformed CUT&Tag using an antibody against 5-Methyl-Cytosine (Supplementary Fig. 2a, b). In general, the 2xMBD2 and 2xMeCP2 constructs recognized the highest number of peaks (around 10,000 each), with approximately 2000 peaks unique to each construct. NTD-MeCP2-IDR and 4xMeCP2 detected ~6800 and ~3600 peaks, respectively. Across all constructs, about 2000 peaks were shared, and roughly 7000 peaks overlapped specifically between 2xMBD2 and 2xMeCP2 (Supplementary Fig. 2d). Plotting the signal on all peaks in a heatmap reveals substantial overlap between all constructs, indicating that the core methylation landscape is consistently captured (Fig. 1b). By contrast, using an antibody against 5-Methyl-Cytosine yielded only 32 detectable peaks, indicating that this approach does not work on nuclei (Supplementary Fig. 2b, d).

Visual inspection confirmed clear enrichments in highly methylated regions as compared to public whole-genome bisulfite sequencing data on human embryonic stem cells^18^ (Fig. 1c), omitting CpGs with high accessibility as measured by ATAC-Seq and lacking DNA methylation (Supplementary Fig. 2b).

To establish biological relevance, we compared C^me^CUT&Tag signals with published whole-genome bisulfite sequencing (WGBS^18^), as well as MBD-Seq^19^ and 5mC-MeDIP^20^ data using ChromHMM analysis^21^, a pre-trained model that categorizes experimentally measured enrichments into distinct chromatin states. The MBD-Tn5 fusion proteins preferentially bound regions exhibiting high DNA methylation ( > 75%) in both reduced-representation^5^ and genome-wide bisulfite sequencing^18^. These regions showed the greatest overlap with transcribed regions, enhancers, and exons, consistent with known DNA methylation enrichment in gene bodies^22^ (Fig. 1d). We used ChIP-Seeker^23^ to further characterize genomic regions showing high enrichment of C^me^CUT&Tag signal and confirmed the enrichment in gene bodies and promoters (Supplementary Fig. 2e). The signal enrichment of C^me^CUT&Tag, as measured by ChromHMM, showed high similarity with similar technologies using 5mC antibodies or MBD-domains to perform Chromatin-Immunoprecipitation (Fig. 1d). We further validated our ChromHMM annotation with CUT&Tag measurements of histone modifications from iPSCs, ATAC-Seq and a Tn5 coupled to the CXXC-domain of MLL1 that recognizes unmethylated CpG island (unC^me^CUT&Tag, Fig. 1d and Supplementary Fig. 2f–i).

Quantitative analysis revealed that MBD-fusion proteins recognize regions with CpG methylation between 40–100%^18^ (Fig. 1e), contrasting sharply with histone modification peaks, which showed no CpG methylation enrichment except for H3K9me3^24,25^ (Fig. 1e and Supplementary Fig. 2j). CpG islands show a bimodal distribution in DNA-methylation signal, with some loci being lowly methylated, as expected, and others being highly methylated (Fig. 1e). Our method reliably captures highly methylated CpG islands, potentially relevant in disease^26^, where hypermethylated CpGs in promoter regions serve as biomarkers.

Based on superior binding affinity, correlation with bisulfite sequencing, higher dynamic range, and peak detection, we selected 2×MBD2-Tn5 for detailed characterization (Fig. 1f, Supplementary Fig. 2c, d, Supplementary Data 1 contains all metadata)^14^. First, we carefully titrated the amount of Tn5 and determined the optimal number of nuclei for subsequent experiments. We determined 600 ng of Tn5 on 50–200 k nuclei as the optimal starting amount (Supplementary Fig. 3a, b).

### Methylation profiling on isolated DNA with C^me^CUT&Tag

**Fig. 2:**
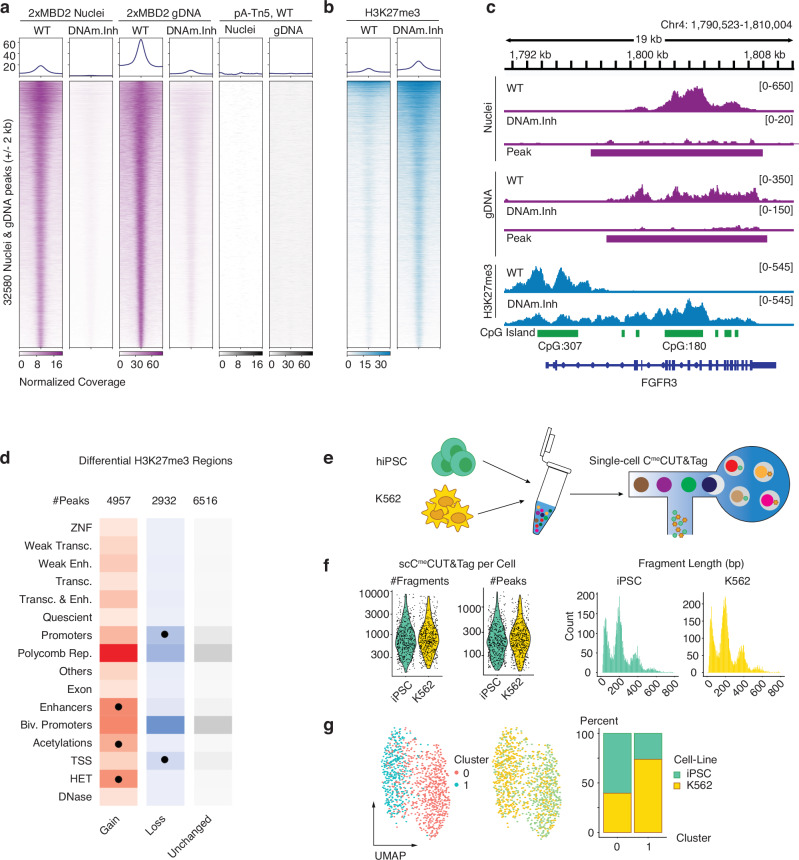
C^me^CUT&Tag is specific to DNA methylation. **a** Heatmaps showing reduced 2xMBD2 binding in human iPSCs treated with a DNMT1 inhibitor, in both native nuclei and purified genomic DNA, confirming the methylation specificity of 2xMBD2 (signal average of 2 replicates). Tagmentation of genomic DNA or nuclei with an untargeted Tn5 does not recapitulate this pattern. **b** Heatmaps of H3K27me3 signal plotted on the 2xMBD2 peaks on both genomic DNA and nuclei (signal average of 2 replicates). Loss of DNA methylation leads to an increase in H3K27me3 signals on 2xMBD2-bound regions. **c** Representative regions illustrating reduced 2xMBD2 binding after DNMT1 inhibitor treatment, with concurrent spreading of H3K27me3 into previously 2xMBD2-bound CpG islands. BigWig signals are scaled individually for visualization. **d** ChromHMM model analysis of the differential H3K27me3 binding sites (FDR < 0.05 and absolute fold change >1.2) upon DNA methylation inhibition. Showing that H3K27me3 is mostly gained in Heterochromatin and Enhancers, and lost at TSS and Promoters. Changes are indicated by a dot. See Supplementary Fig. 5f and Source Data for gene ontology terms on the differential regions. **e** Schematic of the single-cell C^me^CUT&Tag experiment performed on a mixed population of iPSCs and K562 using the 10x Genomics microfluidics platform. Cell lines were prepared independently and mixed during the nuclei isolation. One single-cell suspension was processed for the experiment. **f** Violin Plot showing number of fragments and number of peaks per cell. Histogram showing the fragment length distribution for iPSC and K562 cell lines. **g** K-nearest neighbors (KNN) clustering of the UMAP of scC^me^CUT&Tag data separates the cells into two clusters (right) (Each cluster contains an average number of 1058 and 1356 fragments that passed filters, respectively). Same clustering colored by cell lines iPSC (green—550 cells) and K562 cells (yellow—582 cells). Bar plot quantifying the demultiplexing results for SNPs and the cluster composition (left).

We evaluated whether 2×MBD2-Tn5 functions on isolated genomic DNA, thereby expanding its potential applications to archived samples and compromised chromatin. C^me^CUT&Tag performed efficiently on isolated DNA (Supplementary Fig. 3c–k), and we determined 600 ng of Tn5 on 5 to 50 ng of DNA as the optimal range (Supplementary Fig. 3f, g). The methylation enrichment signals showed high overlap with those from intact nuclei (Supplementary Fig. 3h–k). In general, we observed a stronger signal on isolated DNA, with approximately 25,000 peaks compared to ~10,000 peaks in nuclei. Of these, 2800 peaks are unique to nuclei, whereas 17,800 are unique to isolated DNA (Supplementary Fig. 3h). This difference likely reflects increased Tn5 sensitivity on purified DNA due to reduced steric hindrance compared to chromatinized templates. Nevertheless, plotting the signals in a heatmap reveals substantial overlap between the two conditions, indicating that the core methylation landscape is consistently captured (Fig. 2a). The binding profile of DNA methylation from isolated DNA is also comparable to previously established technologies (MeDIP, MBD-Seq) relying on pulldowns and classical library preparation involving end repair and adapter ligation (Supplementary Fig. 4a–e).

### DNMT1 inhibition confirms C^me^CUT&Tag specificity

To rigorously test the specificity of the observed signals, we treated iPSCs with GSK3484862, a selective DNMT1 inhibitor^27^. This chemical genetics approach specifically reduces DNA methylation, and treatment resulted in substantial decreases in C^me^CUT&Tag enrichment (Fig. 2a) on nuclei as well as isolated genomic DNA, confirming that detected signals reflect genuine DNA methylation. Untargeted pA/G-Tn5 showed no specific enrichments on either gDNA or chromatinized templates, establishing that fusion proteins maintain specificity across different substrates (Fig. 2a). Residual C^me^CUT&Tag signal can be attributed to remaining DNA methylation, as confirmed by both EM-Seq and immunofluorescence analysis. Overall, treatment with GSK3484862 reduced global DNA methylation from 80% to 20%^27^ (Supplementary Fig. 5a, b). Residual peaks also exhibit high CpG content, indicating that the observed binding likely occurs at genuinely methylated regions rather than reflecting nonspecific transposition (Supplementary Fig. 5c, d).

We also profiled H3K27me3 to demonstrate the utility of 2×MBD2-Tn5 for studying relevant changes in the epigenetic landscape and the crosstalk among different epigenetic pathways. We found an increase in H3K27me3 at loci depleted in DNA methylation (Fig. 2b)^28–30^, often coinciding with CpG islands (Fig. 2c and Supplementary Fig. 5e). When we analyzed the genome-wide distribution of H3K27me3, we found that H3K27me3 increased at Polycomb-responsive regions upon DNA methylation depletion, as well as many enhancers and promoters (Fig. 2d) associated with morphogenesis and regulation of membrane potential (Supplementary Fig. 5f, Source Data).

### C^me^CUT&Tag is compatible with single-cell workflows

Leveraging the fact that MBD2-Tn5 can be successfully used in situ within intact nuclei, we sought to test its compatibility with single-cell genomics workflows. To this end, we tagmented nuclei from K562 cancer cells and CAU iPS cells using the C^me^CUT&Tag protocol and processed them through the 10x Genomics single-cell ATAC-seq workflow to encapsulate individual nuclei (Fig. 2e). We successfully recovered 1132 single cells with an average fragment count of 1160 (median 754) and an average peak count of 284 per cell (Fig. 2f, see the “Methods” for specifics on the filtering). Dimensionality reduction and unsupervised clustering revealed two main populations that broadly correspond to the two input cell types, with enrichment of K562 cells in one cluster and iPS cells in the other (Fig. 2g). While the separation is not complete and reflects the limited information content per cell, these results indicate that C^me^CUT&Tag is compatible with droplet-based single-cell workflows and captures cell type–associated DNA methylation differences, albeit at a coarse level given the current data sparsity. We note that this experiment is a proof of concept, and further optimization will be required to improve signal-to-noise ratios and potentially resolve more subtle differences between closely related cell states.

### Adapting C^me^CUT&Tag for base-pair resolution methylation profiling

**Fig. 3:**
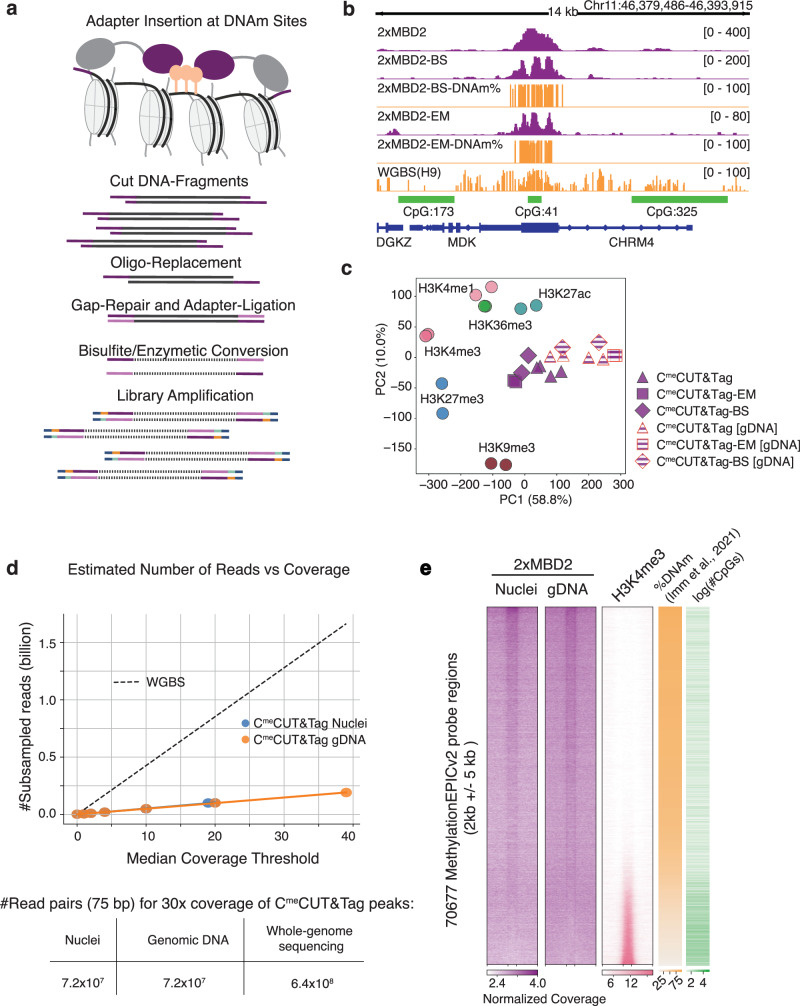
C^me^CUT&Tag is a cost-effective tool for the specific detection of DNA methylation loci. **a** Schematic of the C^me^CUT&Tag protocol followed by bisulfite (BS) or enzymatic (EM) conversion for base-pair resolution methylation profiling. **b** Genome browser view at the *CHRM4* locus comparing 2xMBD2-C^me^CUT&Tag and 2xMBD2-C^me^CUT&Tag-BS/EM on nuclei. CpG methylation profiles from C^me^CUT&Tag-BS and C^me^CUT&Tag-EM closely resemble WGBS profiles at highly methylated CpG loci (*n* = 2, showing one representative replicate each). **c** Principal component analysis (PCA) plot of C^me^CUT&Tag, C^me^CUT&Tag-BS, C^me^CUT&Tag-EM, and histone modification CUT&Tag signals (log2 transformed) on all C^me^CUT&Tag peaks. Bisulfite and enzymatic conversions retain the original binding specificity (for each experiment, 2 replicates are shown). **d** Coverage requirement of C^me^CUT&Tag and WGBS at the C^me^CUT&Tag peaks. C^me^CUT&Tag achieves up to 90% reduction in sequencing cost by selectively enriching for highly methylated regions. **e** Heatmap of 2xMBD2 binding (in both nuclei and genomic DNA) and H3K4me3 over MethylationEPIC array probes (*n* = 2, showing one representative replicate each). A total of 517,789 probes were averaged and merged within a 2-kb window, resulting in 70,676 regions. Regions were then sorted by average beta value (DNA methylation detected in human iPSCs^18^). H3K4me3 is enriched at CpG-dense but lowly methylated regions, while C^me^CUT&Tag preferentially targets CpG-sparse but highly methylated regions.

While C^me^CUT&Tag efficiently identifies highly methylated regions, certain applications require base-pair resolution. We developed a hybrid approach performing bisulfite conversion or Enzymatic Methyl-Seq (EM-Seq) on C^me^CUT&Tag-enriched libraries, combining targeted enrichment with single-nucleotide detection (Fig. 3a). Bisulfite and enzymatic conversion of libraries from both nuclei and genomic DNA yielded signal distributions nearly identical to unconverted libraries (Fig. 3b, c and Supplementary Fig. 6a, b), indicating no substantial bias introduction.

Traditional genome-wide bisulfite sequencing requires 800 million to 1 billion read pairs to cover the human genome at 30× coverage, while C^me^CUT&Tag and C^me^CUT&Tag followed by bisulfite or enzymatic conversion reduce this to 20–100 million reads by limiting coverage to methylated regions, resulting in a 10–40-fold cost reduction (Fig. 3d).

Benchmarking against the Infinium MethylationEPIC array, a clinical tool limited to ~850,000 pre-selected CpG sites, revealed strong signal overlap with high-intensity loci (Fig. 3e)^18^. Undetected loci typically exhibited high H3K4me3 signals and low methylation levels, confirming expected inverse relationships between promoter activity and methylation (Fig. 1e).

### C^me^CUT&Tag maps dynamic methylation in brain organoid development

**Fig. 4:**
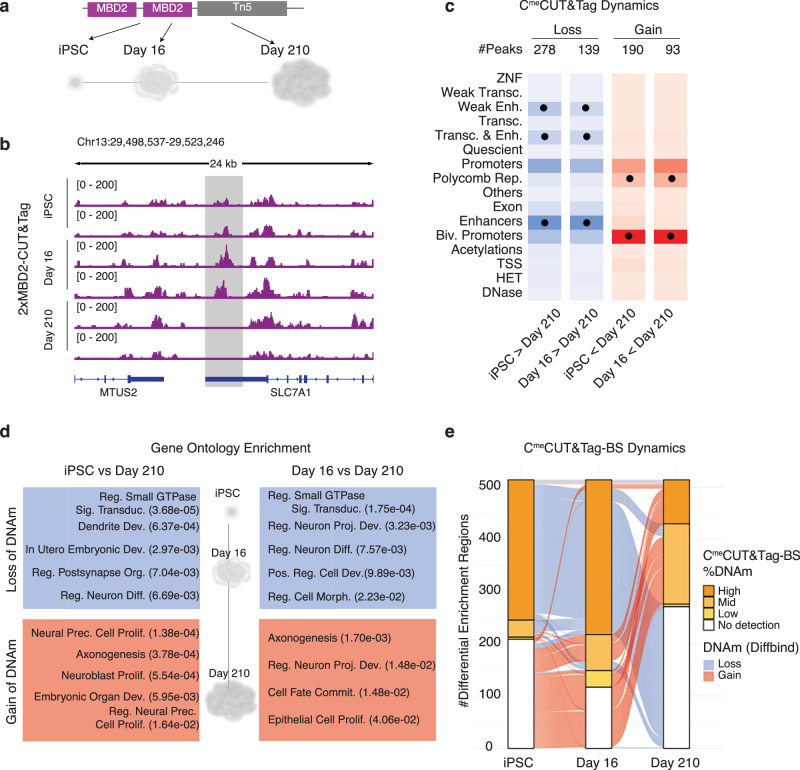
C^me^CUT&Tag monitors the dynamics of brain organoid development. **a** Schematic of the experimental outline. MBD binding profiles were recorded at different stages of brain organoid development. **b** A representative example of a dynamic DNA-methylation peak in organoid development in the genome browser (*n* = 2). **c** ChromHMM model on the gained and lost peaks (FDR < 0.05, source data are provided in the Source Data file) throughout organoid development, showing loss of DNA methylation at enhancers and gain of DNA methylation at bivalent promoters. Dots indicate the most prominent changes. **d** GO annotation and q.values of the genes close to the lost and gained peak set, revealing an increase of DNA methylation at genes regulating progenitor proliferation at late stages and the loss of DNA methylation at genes regulating embryonic development and neural processes during early stages. (Diff. Differentiation, Reg. Regulation, Dev. Development, Sig. Signal, Transd. Transduction, Prec. Precursor, Prolif. Proliferation, Commit. Commitment, Morph. Morphogenesis, Proj. Projection, Pos. Positive). **e** Sankey plot characterizing the dynamic behavior of DNA-methylation at the differential peak set quantified by C^me^CUT&Tag-BS.

To demonstrate its utility for biological processes, we applied C^me^CUT&Tag to human brain organoid development, a process characterized by methylation remodeling during neural differentiation^18,31^. We differentiated iPSCs into multi-region brain organoids, collecting samples at day 16 (primarily neuroepithelium) and day 210 (mixed neuronal populations with emerging astrocytes) (Fig. 4a and Supplementary Fig. 7a). Differential peak analysis revealed systematic methylation changes during neural development (Fig. 4b and Supplementary Fig. 7b). ChromHMM analysis indicated methylation was primarily lost at enhancers and gained at bivalent promoters (Fig. 4c), with affected loci enriched for neuronal development pathways (Fig. 4d).

Quantification using bisulfite-converted C^me^CUT&Tag libraries revealed successive CpG methylation gains from iPSCs to day 210 brain organoids, accompanied by sharp decreases in highly methylated regions between day 16 and day 210 (Fig. 4e). This demonstrates the method’s ability to capture complex, bidirectional methylation dynamics. We benchmarked the DNA-methylation dynamics against published WGBS data^18^ (Supplementary Fig. 7c).

### C^me^CUT&Tag enables DNA methylation-based tumor classification

Being able to capture developmental dynamics prompted us to test whether C^me^CUT&Tag could also be applied to brain tumor classification, which is based on DNA methylation profiling^3^. We analyzed 24 adult brain tumor biopsies, including meningioma, schwannoma, glioblastoma, diffuse midline glioma, pineal parenchymal tumor, and ependymoma.

**Fig. 5:**
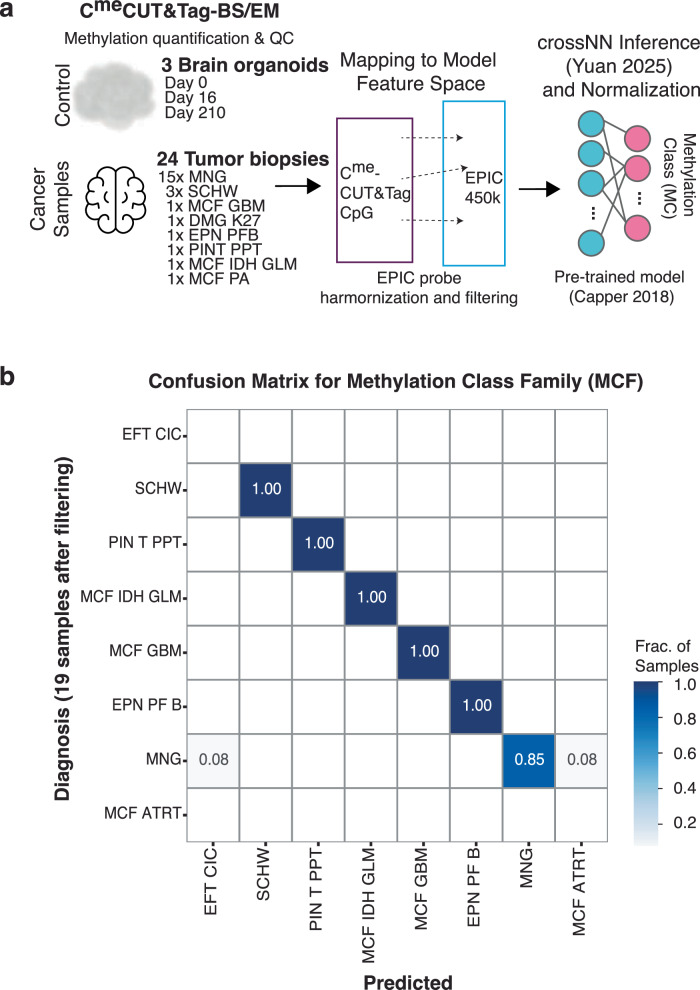
C^me^CUT&Tag- EM methylation profiling enables low-cost CNS tumor classification. **a** Schematic of the prediction pipeline. Brain tumor biopsies were profiled by C^me^CUT&Tag-BS/EM, harmonized to model feature space, and classified using a crossNN model^32^ pre-trained on 2801 reference EPIC 450k samples^3^. **b** Confusion matrix summarizing predicted methylation class families (MCF) for primary biopsies (MNG – Meningioma, *n* = 11; SCHW – Schwannoma, *n* = 2; MCF IDH GLM – Glioma IDH mutant, *n* = 1; EPN PF B – Ependymoma posterior fossa group B, *n* = 1; MCF GBM – Glioblastoma IDH wildtype, *n* = 1; PIN T PPT – Pineal parenchymal tumor, *n* = 1). Labels summarize the fraction of samples that were correctly predicted.

We first projected the C^me^CUT&Tag data into the same feature space used for standard EPIC array profiles of tumor biopsies^3^ and retained only samples with sufficient overlap in shared features (19 samples in total; Fig. 5a). Using crossNN^32^, we then performed tumor classification. Based on C^me^CUT&Tag profiles, we correctly assigned the methylation class family for 17 of the 19 tumors (Fig. 5b).

### Cross-species DNA methylation profiling with C^me^CUT&Tag

Lastly, we validated the cross-species applicability of human MBD2-Tn5 by testing its ability to recognize complex DNA methylation patterns in both isolated DNA and intact nuclei from a non-mammalian vertebrate model. Using 22 h post-fertilization (hpf) zebrafish embryos, we successfully profiled genome-wide DNA methylation patterns using C^me^CUT&Tag (Supplementary Fig. 8a–c). We benchmarked these data against previously published MethylCap and whole-genome methylation datasets from zebrafish embryos^33,34^ (Supplementary Fig. 8b–d). Genome-wide clustering revealed strong concordance between MethylCap and C^me^CUT&Tag signals. Moreover, peaks identified by both methods showed elevated DNA methylation levels and CG content compared to shuffled control regions and displayed highly similar genomic distributions, as assessed using ChIPseeker (Supplementary Fig. 8e).

## Discussion

C^me^CUT&Tag addresses critical limitations in DNA methylation detection by dramatically reducing costs while maintaining quantitative accuracy compared to WGBS or single-molecule direct methylation detection technologies such as PacBio or Oxford Nanopore (Supplementary Data 2). The 10–40 fold reduction in sequencing requirements transforms methylation analysis from a specialized, expensive technique to an accessible tool for routine investigation. The method’s compatibility with both intact chromatin in situ and isolated DNA, combined with optional bisulfite conversion or EM-Seq, provides flexibility for diverse applications. Other conversion workflows like TET-assisted pyridine borane sequencing (TAPS) could also be integrated in the future^35^.

C^me^CUT&Tag builds upon earlier enrichment-based methods such as MethylCap-seq^7^ and MiGS^8^ by integrating the DNA methylation-binding domain directly with Tn5 transposase. This direct fusion eliminates the need for multi-step library preparation involving end repair, adapter ligation, and purification, substantially reducing processing time and reagent cost. Moreover, the fusion enables efficient adapter integration in situ, directly within chromatin, without the need for DNA extraction or fragmentation. As a result, C^me^CUT&Tag is not only faster and more cost-efficient, but also lowers input requirements, enabling profiling from as few as thousands of nuclei (50 k) or nanogram-level DNA (5 ng) (Supplementary Data 2 contains a comparison of all methods).

Here, we focused on testing the method with single-cell sequencing workflows. In-situ digestion allows integration with microfluidic-based single-cell technologies, as demonstrated here, or split-pool barcoding-based approaches, and can substantially increase throughput and cell numbers compared to plate-based single-cell DNA methylation mapping technologies. Our technology could also be compatible with Tn5-based spatial genomics workflows^36^. Limitations of C^me^CUT&Tag include that, in its current setup, it cannot discriminate between 5mC and 5hmC and cannot cover regions with methylation levels below 40%. Our demonstration of dynamic methylation profiling during brain organoid and zebrafish development, as well as for tumor subtype classification, illustrates the method’s potential for advancing understanding of epigenetic regulation in development and disease. The cost-effectiveness and scalability of C^me^CUT&Tag make it particularly suitable for large-scale studies, clinical applications, and research programs that require methylation profiling across multiple conditions. This approach represents a step towards broadening access to genome-wide DNA methylation analysis.

## Methods

### Cloning and generation of Tn5 fusion proteins

MBD domains were amplified from organoid cDNA or the respective cDNA clone of the ORFeome Collaboration (OC) (MeCP2 AM392557/EU17665)^37^ or directly synthesized by TwistBiosciences and subsequently cloned into TXB1-pA/G-Tn5^10^ using ClaI/EcoRI or NcoI/EcoRI restriction digest, followed by ligation or Gibson assembly. For the generation of double domain constructs, the individual domains were fused by PCR and connected by a flexible linker. To integrate multiple domains, a silent mutation was introduced in the flexible linker to generate a BamHI site, and the additional domains were inserted by Gibson assembly.

#### 2xMBD2 (flexible linker)

FZ243_NcoI_Flag_MBD2_fw ccatgggtGATTACAAGGATCACGATGGCGATTACAAGGATCACGATATCGATTACAAGGATGATGATGATAAGatgaccatgattacgccaGAGAGCGGGAAGAGGATGGATTGCCCG

FZ245_BamHI_MBD2-linker_rev cctccactggatccgccacctccCATCTTTCCAGTTCTGAAGTCAAAAC

FZ246_BamHI_linker_MBD2_fw ggaggtggcggatccagtggaggtggcggaagcagtGAGAGCGGGAAGAGGATGGATTGC

FZ244_EcoRI_SV40_MBD2_rev gaattctttatcgtcatcgaccttccgcttcttctttggCATCTTTCCAGTTCTGAAGTCAAAAC

#### NTD-MeCP2-IDR

FZ248_NcoI_MeCP2-NTD_fw ccatgggtGATTACAAGGATCACGATGGCGATTACAAGGATCACGATATCGATTACAAGGATGATGATGATAAGatgaccatgattacgccaATGGTAGCTGGGATGTTAGGGCTCAGGG

FZ249_EcoRI_MeCP2-ID_revgaattctttatcgtcatcgaccttccgcttcttctttggACCCTCTGACGTGGCCGCCTTGGG

#### 2xMeCP2 (flexible linker)

NcoI_MeCP2_fwccatgggtGATTACAAGGATCACG

BamHI_MeCP2linker_rev cctccactggatccgccacctccCTCTCGCCGGGAGGGGCTCCCTCTC

BamHI_linker_MeCP2_fw ggaggtggcggatccagtggaggtggcggaagcagtGACCGGGGACCCATGTATGATGACC

EcoRI_MeCP2_revgaattctttatcgtcatcgaccttcc

#### 4xMeCP2 (flexible linker)

FZ250_MeCP2_gib_assembly_fw TCCCGGCGAGAGggaggtggcggaGGATCTagtggaggtggcggaagcagtGAC

FZ251_MeCP2-MBD2_gib_assembly_rev CactgcttccgccacctccactggaTCCtccgccacctccCTCTCGCCGGGAGGGGCTCC

#### 2xMLL1-CXXC (flexible linker)

CXXC-MLL1_part1 GTTTAACTTTAAGAAGGAGATATACCATGGGTGATTACAAGGATCACGATGGCGATTACAAGGATCACGATATCGATTACAAGGATGATGATGATAAGATGACCATGATTACGCCAAAGAAAGGACGTCGATCGAGGCGGTGTGGGCAGTGTCCCGGCTGCCAGGTGCCTGAGGACTGTGGTGTTTGTACTAATTGCTTAGATAAGCCCAAGTTTGGTGGTCGCAATATAAAGAAGCAGTGCTGCAAGATGAGAAAATGTCAGAATCTACAATGGATGCCTTCCAAAGGAGGTGGCGGATCCAGTGGAGGTGGCGGAAGCAGT

CXXC-MLL1_part2 GGAGGTGGCGGATCCAGTGGAGGTGGCGGAAGCAGTAAGAAAGGACGTCGATCGAGGCGGTGTGGGCAGTGTCCCGGCTGCCAGGTGCCTGAGGACTGTGGTGTTTGTACTAATTGCTTAGATAAGCCCAAGTTTGGTGGTCGCAATATAAAGAAGCAGTGCTGCAAGATGAGAAAATGTCAGAATCTACAATGGATGCCTTCCAAAGATGACGATAAAGAATTCGGTGGCGGTGGCTCTGGCGGTGGTGGGAGTGGAGGTGGGGGATCAGGAGGAGGCGGTTCCCATATGATTACCAGTGCACTGCATCGT

### Purification of Fusion proteins

After sequencing the final constructs, plasmids were transformed into Rosetta cells.

The bacteria were grown in 400 ml of LB supplemented with Ampicillin and Chloramphenicol to an OD600 of 0.4-0.6. Expression was induced with 0.25 mM IPTG, and the protein was expressed at 18 °C overnight. Cells were harvested, and pellets were stored at −80 °C until further processing. The purification was performed on Chitin resin (New England Biolabs, #S6651S) as described^38^, with small modifications. The cells were lysed using a Fisherbrand sonicator for 2.5 min, 10/10/ on/off with intensity at 70%.

After dialysis and concentration using Amicon Ultra-4 Centrifugal filters (Millipore, #UFC803024), the protein was diluted to 50% glycerol and a final concentration of 300–400 ng/µl determined by Bradford and through the intensity of the band on a gel, and loaded with adapters or methylated adapters before use (below). CXXC-MLL1 is not stable for long-term storage in glycerol and should be used only immediately after purification.

Tn5MErev [phos]CTGTCTCTTATACACATCT

Tn5ME-A TCGTCGGCAGCGTCAGATGTGTATAAGAGACAG

Tn5ME-B GTCTCGTGGGCTCGGAGATGTGTATAAGAGACAG

### Culture for cancer cells, iPSCs, and organoids

Cell lines used in the study were derived from different sources:

WIBJ2 (WTSli046-A, female) and HOIK (HPSI0314i-hoik_1, female) HipSci resource^39^ HCNP NeuroNA foundation,

CAU (female) HCNP NeuroNA foundation, Phenocell PC-1505

K562 (chronic myeloid leukemia in blast crisis), DSMZ ACC 10

For culturing, iPS cells were grown on Matrigel (Corning, #354277) coated 6-well dishes in mTeSR Plus (StemCell Technologies, #1000276) supplemented with penicillin/streptomycin (P/S, 1:200, Gibco, #15140122). To propagate the cells, they were dissociated with TrypLE (Gibco, #12605010) or EDTA in DPBS (final concentration 0.5 mM) (Gibco, #15575020) and kept on Rho-associated protein kinase (ROCK) inhibitor Y-27632 (final concentration 5 μM, StemCell Technologies, #72302) for one day. Cells were stored in liquid nitrogen in mFreSR (StemCell Technologies, #05855) and tested for mycoplasma (Venor GeM Classic, Minerva Biolabs) after each thawing cycle.

To generate brain organoids, cells were grown to a confluency of approximately 50% and then dissociated using TrypLE. 2000–3000 cells were aggregated in 96-well ultra-low attachment plates (Corning, #CLS7007) to form embryoid bodies (EBs). We followed an unguided protocol to obtain brain organoids^40^, with a few modifications. EBs were aggregated and cultured in mTeSR Plus, and neural induction medium was added when the EBs had reached a diameter of approximately 400–500 µm (usually on day 5). Retinoic acid-containing neural differentiation medium was only added from day 40 onward^41^. Cerebral organoids were grown shaking in 6 cm dishes until processing for experiments.

K562 cells were cultured in RPMI with 10% FCS (Sigma, SLM-240-B) supplemented with penicillin/streptomycin (P/S, 1:200, Gibco, #15140122).

Cell lines used in the study were authenticated by comparing single-nucleotide polymorphisms identified from single-cell RNA-Seq and CUT&Tag to reference datasets.

### DNMT1 inhibitor treatment

For inhibitor treatment, WIBJ2 cells^39^ were grown to a confluency of 30% in mTESR Plus as described. Following GSK3484862 was added to the medium at a final concentration of 5 µM. Cells were cultured and split regularly for two more weeks to ensure the depletion of DNA methylation.

### Preparation of single-cell suspensions

Human brain organoids were dissociated into single-cell suspensions using the Neural Tissue Dissociation Kit (P) (Miltenyi Neural Dissociation protocol)^10^. Organoids were cut into pieces using a scalpel, thoroughly washed in DPBS supplemented with 0.5% BSA, and incubated with papain-containing Enzyme Mix 1 at 37 °C for 15 min, followed by DNase-containing Enzyme Mix 2. Mechanical dissociation was performed by sequential trituration using P1000 and P200 pipette tips with intermittent 37 °C incubations until a homogeneous suspension was obtained. Cells were filtered through a 30 μm strainer, pelleted at 300 × g for 5 min, resuspended in wash buffer, and counted using Trypan Blue.

Zebrafish embryos (wild-type Tupfel longfin/AB) were cultured and dissociated^42^. Briefly, embryos were collected 20 min post-fertilization and cultured in E3 embryo medium at 28 °C until the desired developmental stage (H22). Embryos were extensively washed in E3 medium, transferred to agarose-coated dishes, and dechorionated with pronase and washed again. For cell dissociation, embryos were deyolked in chilled buffer and mechanically dissociated by pipetting to obtain single-cell suspensions. For nuclei isolation, cell pellets were lysed in detergent-containing buffer, washed, and resuspended in CUT&Tag Wash Buffer.

### CUT&Tag for histone modifications

Starting with 0.1-1 Mio cells after dissociation, cells were transferred into CUT&Tag wash buffer (20 mM HEPES [pH 7.5] (Jena Bioscience, #CSS-511), 150 mM NaCl (Sigma Aldrich, #S6546), 0.5 mM Spermidine (Sigma Aldrich, #S0266), 5 mM sodium butyrate (Sigma Aldrich, #303410), Roche Protease Inhibitor (Sigma Aldrich, #11873580001). Following 15 µl of BioMag ConcanavalinA beads (Polysciences, #86057-3) in binding buffer (20 mM HEPES (pH 7.5), 10 mM KCl, 1 mM CaCl_2_, 1 mM MnCl_2_) were added to the sample and incubated on the wheel for 15 min at RT. Subsequently, the cells were collected on a magnet and lysed by adding CUT&Tag wash buffer supplemented with 0.01% Digitonin. Lysis was monitored under a microscope with Trypan Blue staining. After lysis was complete, the nuclei were washed again with the CUT&Tag wash buffer. If possible, all samples were split, and H3 or another chromatin mark CUT&Tag was performed on the same starting material to be used as a normalizer. The antibody (1 µg per reaction, against histone modifications or 5mC) was added together with 2 mM EDTA final, and the sample was incubated on a rocking platform at 4 °C overnight. The next day, the samples were washed once with CUT&Tag wash buffer, and the secondary antibody was added to the reaction, which was then incubated for 1 h at 4 °C on a rocking platform. After two additional washes the Tn5 (pA/G-Tn5) was added (600 ng per reaction) in CUT&Tag med buffer (20 mM HEPES [pH 7.5] (Jena Bioscience, #CSS-511), 300 mM NaCl (Sigma Aldrich, #S6546), 0.5 mM Spermidine (Sigma Aldrich, #S0266), 5 mM sodium butyrate (Sigma Aldrich, #303410), Roche Protease Inhibitor (Sigma Aldrich, #11873580001)). Tn5 was allowed to bind for 1 h at 20 °C on a rocking platform. After two additional washes, the cutting was induced through the addition of 10 mM MgCl_2_ in the CUT&Tag med buffer. After 1 h at 37 °C, the reaction was stopped by adding a final concentration of 20 mM EDTA, 0.5% SDS, and 10 mg Proteinase K. The reaction was then incubated at 55 °C for 30 min and finally inactivated at 70 °C for 20 min.

The DNA fragments were purified using the ChIP DNA Clean & Concentrator kit (Zymo Research, #D5205). For elution from the columns, 10 pg of Tn5-digested and purified lambda DNA (New England Biolabs, #N3011S) were added as a spike-in normalizer for later analysis.

Overview of the antibodies used in the study:

5mC Diagenode C1520003, RD-007

H3K27ac Diagenode C15410196, A1723-0041D

H3K27me3 Diagenode C15410195, A0824D

H3K36me3 Abcam AB9050, 1063779-1

H3K4me1 DiagenodeC15410194, A1862D

H3K4me3 DiagenodeC15410003, A1052D

H3K9me3 Abcam ab176916, GR3218257-2

### C^me^CUT&Tag on gDNA

Following dissociation of organoids or tissues, genomic DNA was extracted using the DNeasy Blood and Tissue Kit (Qiagen, #69504) according to the manufacturer’s instructions. DNA methylation profiling was performed using an MBD-fused Tn5 transposase to selectively target methylated DNA. C^me^CUT&Tag for tumor biopsies was performed on surplus DNA from fully anonymized tissues. Between 1 and 50 ng of purified genomic DNA was incubated in 100 µl CUT&Tag med buffer (20 mM HEPES, pH 7.5; 300 mM NaCl; 0.5 mM spermidine; 5 mM sodium butyrate; Roche protease inhibitor) supplemented with 600 ng of the indicated Tn5 transposase construct (see Supplementary Data 1 for individual sample information). Binding reactions were carried out at 4 °C for 2 h. ProteinA/G coupled Tn5 (pA/G-Tn5) was used as a control and treated in the same way.

Tagmentation was initiated by the addition of MgCl₂ to a final concentration of 10 mM in CUT&Tag med buffer. Reactions were incubated at 37 °C for 1 h and terminated by the addition of EDTA to a final concentration of 12.5 mM.

DNA fragments were purified using the ChIP DNA Clean & Concentrator kit (Zymo Research, #D5205). For normalization during downstream analysis, 10 pg of Tn5-digested and purified lambda DNA (New England Biolabs, #N3011S) was added to the elution buffer during column elution.

### C^me^CUT&Tag on nuclei

Starting from 0.1–1 Mio. cells after dissociation (see Supplementary Data 1 for individual sample information), cells were transferred into CUT&Tag wash buffer (20 mM HEPES, pH 7.5; 150 mM NaCl; 0.5 mM spermidine; 5 mM sodium butyrate; Roche protease inhibitor cocktail). A total of 15 µl BioMag Concanavalin A beads (Polysciences, #86057-3), pre-equilibrated in binding buffer (20 mM HEPES, pH 7.5; 10 mM KCl; 1 mM CaCl₂; 1 mM MnCl₂), were added, and samples were incubated on a rotating wheel for 15 min at room temperature.

Cells were collected using a magnetic stand and lysed by incubation in CUT&Tag wash buffer supplemented with 0.01% Digitonin. Lysis efficiency was monitored by Trypan Blue staining under a light microscope. Following complete lysis, nuclei were resuspended in 150 µl CUT&Tag med buffer (20 mM HEPES, pH 7.5; 300 mM NaCl; 0.5 mM spermidine; 5 mM sodium butyrate; Roche protease inhibitor).

MBD-fused or CXXC-fused Tn5 transposase was added, and binding reactions were carried out at 4 °C for 2 h. ProteinA/G coupled Tn5 (pA/G-Tn5) was used as a control and treated in the same way. Beads were washed twice with 400 µl CUT&Tag med buffer, after which 100 µl CUT&Tag med buffer supplemented with 10 mM MgCl₂ was added to the beads to initiate tagmentation. Reactions were incubated at 37 °C for 1 h and terminated by the addition of EDTA (12.5 mM final), SDS (0.5% final), and Proteinase K (10 mg/ml final). Samples were incubated at 55 °C for 30 min, followed by enzyme inactivation at 70 °C for 20 min.

DNA fragments were purified using the ChIP DNA Clean & Concentrator kit (Zymo Research, #D5205). For normalization during downstream analysis, 10 pg of Tn5-digested and purified lambda DNA (New England Biolabs, #N3011S) was added to the elution buffer during column elution.

### Generation of sequencing libraries for CUT&Tag and C^me^CUT&Tag

Purified fragments were indexed for 15 cycles (1 × 5 min at 58 °C, 1 × 5 min at 72 °C, 1 × 30 s at 98 °C, 14 × 10 s at 98 °C, 30 s at 63 °C, 1 × 1 min at 72 °C, ∞ at 4 °C) using NEBNext HighFidelty 2x PCR Master Mix (New England Biolabs, M0541S) and Illumina i5 and i7 indices^43^. The libraries were then purified using AmPure beads (Beckman Coulter, #A63881). They were measured and quality controlled with the Qubit DNA HS Assay (Thermo Scientific, #Q32854) and analyzed on the TapeStation (Agilent, #5067-4626). The libraries were then sequenced (PE, 2 × 75 bp).

### DNA methylation C^me^CUT&Tag on single nuclei

Starting from 2 Mio. cells following dissociation, cells were transferred into wash buffer (20 mM HEPES, pH 7.5; 300 mM NaCl; 0.5 mM spermidine; 5 mM sodium butyrate; 1× Roche protease inhibitor cocktail; 2% BSA). Cells were lysed by incubation in wash buffer supplemented with 0.01% digitonin. Lysis efficiency and the quality of single-nucleus suspensions were monitored by Trypan Blue staining under a light microscope. Samples were centrifuged at 300 × *g* for 5 min at 4 °C using a swing-bucket rotor. Following complete lysis, nuclei were resuspended in 200 µl wash buffer. Equal amounts of nuclei of iPSC (CAU), leukemia cells (K562), and brain organoid cells (HOIK) were mixed.

2 µl of MBD-fused Tn5 transposase was added, and binding reactions were carried out at 4 °C for 2 h. Nuclei were washed twice with 200 µl wash buffer, with centrifugation at 300 × *g* for 5 min at 4 °C between washes. Subsequently, 200 µl tagmentation buffer (wash buffer supplemented with 10 mM MgCl₂) was added to initiate tagmentation. Reactions were incubated at 37 °C for 1 h and terminated by the addition of stop buffer (1× DNB buffer from the Chromium Next GEM Single Cell ATAC Reagent Kits v2; 2% BSA; 25 mM EDTA).

Samples were filtered through a 40-µm Flowmi Tip Strainer (#BAH136800040-50EA) and centrifuged. Nuclei pellets were resuspended in 200 µl 1× DNB buffer supplemented with 2% BSA, and nuclei concentration and single-nucleus integrity were assessed by light microscopy. Nuclei were pelleted again, resuspended in approximately 20 µl buffer, and counted once more prior to GEM generation and barcoding. We obtained around 120 k nuclei at this point after the incubations, corresponding to around 15% of the initial input.

For single-nucleus library preparation, nuclei suspensions were mixed with ATAC buffer (Chromium Next GEM Single Cell ATAC Reagent Kits v2) to a final volume of 15 µl (7 µl ATAC buffer, up to 8 µl nuclei suspension, and 1× DNB buffer with 2% BSA). For the sample, 10k nuclei were loaded onto the Chromium Next GEM Chip and processed following the manufacturer’s protocol (steps 2–4: GEM generation and barcoding, post-GEM incubation cleanup, and library construction). After sequencing and before filtering, we obtained 1950 cells with valid barcodes from the experiment, corresponding to a recovery rate of 20% compared to what had been loaded.

### C^me^CUT&Tag followed by bisulfite or enzymatic conversion

To achieve nucleotide resolution, we performed bisulfite conversion or enzymatic methylation conversion on the Tn5-digested DNA fragments. During chromatin digestion, we used MBD-Tn5 loaded with only one adapter (Tn5ME-A or Tn5ME rev), both fully methylated to preserve adapter integrity during cytosine conversion (see below)^44^.

Tn5mC1.1-A1block /5Phos/CT GTC TCT TAT ACA /3ddC/

Tn5ME-A T[5MedC]GT[5MedC]GG[5MedC]AG[5MedC]GT[5MedC]AGATGTGTATAAGAGA[5MedC]AG

Tn5mC-ReplO1 /5Phos[5MedC]TGT[5MedC]T[5MedC]TTATA[5MedC]A[5MedC]AT[5MedC]T[5MedC][5MedC]GAG[5MedC] [5MedC]CA[5MedC]GAGA[5MedC]/3InvdT/

After the first tagmentation step, we spiked in 10 pg unmethylated T7 DNA to control the conversion efficiency. We purified the reaction using the ChIP DNA Clean & Concentrator kit (Zymo Research, #D5205) and eluted in 12 µl.

To replace the Tn5mC1.1-A1block oligo with the Tn5mC-ReplO1, purified DNA fragments were incubated with 1 mM dNTPs and 1 mM of Tn5mC-ReplO1 1× Ampligase buffer (Lucigen). Reactions were carried out in a thermal cycler using the following program: 1 min at 50 °C, 10 min at 45 °C, followed by cooling to 37 °C at a ramp rate of −0.1 °C s⁻¹. Then 1 µl of T4 Polymerase (M0203S) and 2.5 µl of Ampligase (Lucigen) were added. The reaction was incubated at 37 °C for 30 min. Reactions were terminated by adding EDTA to a final concentration of 25 mM. After purification of the fragments using the ChIP DNA Clean & Concentrator kit (Zymo Research, #D5205), we used either EZ DNA Methylation Lightning Kit (Zymo, D5030-E) or the NEBNext Enzymatic Methyl-seq v2 Conversion Module (New England Biolabs, #E8020) following the manufacturer’s instructions to convert unmethylated Cytosin into Uracil. The DNA was cleaned up on Zymo columns or purified using magnetic bead–based cleanup and eluted in 25 µl EB buffer supplemented with 10 pg of Tn5-digested and purified lambda DNA (New England Biolabs, #N3011S) as a spike-in normalizer for downstream analysis.

### Sequencing libraries after bisulfite and enzymatic conversion

For library amplification, 21 µl of DNA fragments, and 2 µl of each i7 and i5 index primers (25 µM) were added to 25 µl of KAPA HiFi Uracil Mastermix (Roche, #KK2801). The samples were run in the thermocycler for 17× cycles (1 × 45 s at 98 °C, 17 × 15 s at 98 °C, 30 s at 63 °C, 30 s at 72 °C, 1 × 2 min at 72 °C, ∞ at 4 °C).

### Immunofluorescence stainings

After one week of treatment with the DNA methylation inhibitor GSK-3484862 or DMSO, iPSCs (WIBJ2) were split on poly-L-lysine-treated coverslips for immunostaining and allowed to recover for 2 days. Coverslips were fixed with 4% PFA for 15 min at room temperature. Next, coverslips were washed 3 times with PBS. Antigen retrieval was performed for 20 min at 50 °C with preheated HistoVT One (Nacalai, 06380). Following antigen retrieval, the coverslips were permeabilized with 0.1% Tween in PBS. Afterward, the coverslips were incubated with 2 M HCl for 30 min at 37 °C to denature the DNA. The slides were then neutralized with 0.1 M Borate and given a quick wash with PBS. Then, the slides were blocked with PBS + 0.1% Tween + 1% BSA for 1 h at room temperature and incubated with primary antibodies 5mC (Diagenode, C15200081, 1:5000) and H3K27me3 (Diagenode, C15410195, 1:1000) at 4 °C overnight. The next day, the coverslips were washed three times with 0.1%Tween+PBS and incubated with secondary antibodies for 1 h at room temperature. After secondary antibody incubation (anti-mouse 488 (Thermo, A21202), anti-rabbit 488 (Thermo, A10040)), the coverslips were incubated with DAPI diluted in PBS + 0.1% Tween and further washed twice with PBS + 0.1% Tween. Finally, coverslips were mounted in Vectashield. Slides were imaged with a Nikon Ti2 microscope.

### Pre-processing, alignment, and normalization of CUT&Tag data

First, a hybrid genome was generated consisting of the human genome (hg38, Ensembl release 113, primary assembly, https://ftp.ensembl.org/pub/release-113/fasta/homo_sapiens/dna/), Escherichia phage Lambda (https://www.ncbi.nlm.nih.gov/datasets/genome/GCF_000840245.1/), and Escherichia phage T7 (https://www.ncbi.nlm.nih.gov/datasets/genome/GCF_000844825.1/). Hybrid genome indices were built using the createIndices pipeline from snakePipes (version 3.1.0^45^).

CUT&Tag sequencing data were aligned to the hybrid genome using the DNAmapping workflow from snakePipes with the BWA-mem2 aligner^45^. The resulting alignment BAM files were converted to BigWig files using bamCoverage from deepTools^46^ (version 3.3.0) and subsequently scaled based on the number of reads mapped to the spike-in regions. When spike-ins were not included, normalization was omitted, and human-aligned data were used as-is for downstream analysis.

For paired-end sequencing libraries, fragment sizes were assessed using the bamPEFragmentSize tool from deepTools.

### Peak calling and chromatin state annotation of CUT&Tag data

Broad peaks were called from the BAM files of individual sequencing runs using MACS3^47^ (version 3.0.1) with the parameters --broad-cutoff 0.1, --nolambda. The fraction of reads in peaks (FRiP score) was calculated for each BAM file using featureCounts from subread (version 2.0.2^48^), by quantifying reads overlapping the corresponding broad peaks. The consensus peaks of biological replicates were identified using ChIP-R^49^.

### Comparison of MBD constructs

To compare the enrichment patterns of different MBD constructs, a union peak set of C^me^CUT&Tag was generated by combining all consensus peaks across constructs using ChIP-R. This union set represents regions identified by at least one construct. Peak intersections were visualized using Intervene^50^.

To assess the overlap between construct-specific peaks and CpG islands, we obtained the CpG island track for hg38 from the UCSC Genome Browser (http://genome.ucsc.edu) and filtered the BED files to retain only standard chromosomes (chr1-22, X, Y, and M).

### Processing of the published human DNA methylation dataset

Six published DNA methylation datasets and one ATAC dataset were analyzed:

1. A reduced representation bisulfite sequencing (RRBS) dataset^5^ of human embryonic stem (hES) or induced pluripotent stem (iPS) cells. (GEO accession number GSE25970).
2. A whole-genome bisulfite sequencing (WGBS) dataset^18^ of cerebral organoids. (GEO accession numbers GSE82022).
3. A MethylationEPIC BeadChip Kit dataset of human cortical organoids^48^. (GEO accession numbers GSE150122).
4. A MethylationEPIC BeadChip Kit dataset of human iPSC^51^. (GEO accession number GSE158089).
5. A MBD-seq (MBD2 domain) dataset on hESC (H1)^19^ (GEO accession number GSE159071).
6. MeDIP-seq datasets on human fetal brain and neurosphere cultured cells—cortex derived^20^ (GEO accession numbers GSM66910, GSM66912, GSM66914, GSM66915, GSM707019, GSM817248, GSM817249).
7. ATACseq datasets on human iPSCs^52^ (GEO accession number GSE203377).

For the RRBS dataset, raw methylation data (in BED format) were first converted from the hg16 to the hg38 genome assembly using the liftOver tool^53,54^. Biological replicates were merged across cell stages, including 20 hESC lines, 12 hiPSC lines and 5 human embryoid body (hEB) lines at day 16. CpG methylation percentages were then calculated for each cell stage.

For the WGBS dataset, CG methylation profiles were extracted from pre-processed data in the allc format using the methylpy filter-allc command^55^. The resulting allc files were converted to BED format and lifted from hg19 to hg38. Biological replicates were then merged to compute CpG and non-CpG methylation percentages for hESCs, hEBs (day 16), cerebral organoids (days 40 and 60), and fetal cortex (middle frontal gyrus, 19 gestational weeks).

For the two MethylationEPIC BeadChip Kit datasets, the summarized signal file containing methylated and unmethylated intensities was used. Beta values for each probe were calculated with an offset of 100. Probe coordinates (MAPINFO) from the Infinium MethylationEPIC v2.0 Kit (https://emea.illumina.com/products/by-type/microarray-kits/infinium-methylation-epic.html) were used to match probes to genomic locations on the hg38 assembly.

For the MBD-seq datasets^19^, the raw sequencing data for four replicates were processed in the same way as C^me^CUT&Tag described above.

For the MeDIP-seq datasets, the BigWig files were downloaded from NIH Roadmap Epigenomics Project Data Listings^20^. The two BigWig files for cortex-derived neurospheres were averaged, and the five BigWigs for fetal brains were averaged.

For the ATAC-seq data^52^, the peaks (broadPeak format) of 24 iPS cell lines were merged.

### Processing of the published zebrafish DNA methylation dataset

Two publicly available DNA methylation datasets from zebrafish embryos (24 h post fertilization, hpf) were analyzed:

1. Whole-genome bisulfite sequencing (WGBS) data (two biological replicates; GEO accession: GSE179673^34^).
2. MethylCap-seq and H3K27me3 ChIP-seq data (one biological replicate; GEO accessions: GSE35050 and GSE70847^33^).

The zebrafish reference genome (GRCz11) was obtained from Ensembl (release 115; https://ftp.ensembl.org/pub/release-115/fasta/danio_rerio/dna/). All datasets were processed using snakePipes with the same parameters as described above to ensure consistent preprocessing and downstream analysis.

### Chromatin state and ChIPseeker annotation of CUT&Tag peaks

Chromatin states of the consensus peaks were annotated using ChromHMM^21,56^ by mapping the peak BED files to a pre-defined 100-state model using the OverlapEnrichment function. The 100-state model was further summarized based on the provided group annotations.

To annotate the genomic region of the peaks, the annotatePeak function from ChIPseeker^23^ was used with the parameters tssRegion = c(−3000, 3000), annoDb = “org.Hs.eg.db”, overlap = ‘TSS’. Exons, UTRs, and introns were grouped into the gene body. Peak sets were overlapped with CpG islands^7^.

### CG count and methylation profiling of CUT&Tag peaks

To further characterize CUT&Tag binding regions, CG content (%) and CpG dinucleotide counts (case-insensitive) were calculated using the nuc function from bedtools (version 2.30.0^57^).

CpG methylation profiles were derived by intersecting peak regions with the published WGBS^18^ data using the map function in bedtools. For each peak, the average CpG methylation level (%mCpG) was computed as the total methylation signal divided by the number of CpG sites present within the region.

Background peak sets were generated using the shuffle function from bedtools, which randomly relocates genomic intervals while preserving their original length distribution.

To assess how C^me^CUT&Tag enrichment (BigWig signals) varies with methylation levels, peaks were grouped into bins based on their %mCpG values in 10% intervals (0–10%, 10–20%, …, 90–100%). For each bin, the median C^me^CUT&Tag signal across all peaks (computed using bigWigAverageOverBed) was calculated and plotted.

### Differentially enriched H3K27me3 regions upon DNA methylation inhibition

BAM files from H3K27me3 CUT&Tag experiments under DMSO and DNMT1 inhibitor treatment (2 biological replicates per condition) were analyzed using the DiffBind package in R^58^. Regions with a false discovery rate (FDR) < 0.05 were considered significantly differentially enriched. Diffbind regions were then annotated using ChromHMM chromatin state models. Gene ontology analysis of the nearest genes to the differentially bound regions was conducted using the enrichGO function from the clusterProfiler package in R^59^.

### Single-cell C^me^CUT&Tag

Single-cell C^me^CUT&Tag data were mapped to hg38 using cellranger count, resulting in 1950 cells with valid 10x barcodes. After filtering for the number of counts per cell ( > 20), the number of features per cell ( > 20), nucleosome signal ( < 3.5), FRiP score ( > 15), and number of fragments passed filter ( < 10,000), 1799 cells were retained. The average number of fragments passed filtering per cell is 1054 (median 629), comparable to previous reports^10,60^; the average FRiP score is 29%.

To assign individual cells to the different cell lines, Demuxlet^61^ was used for genotype-based SNP demultiplexing using VCF files from K562 (https://www.encodeproject.org/files/ENCFF538YDL/), CAU, and HOIK (built with bcftools). After SNP demultiplexing, 1132 cells with an average of 1160 fragments per cell (median 754) were retained and used for downstream clustering analysis with FindNeighbors and FindClusters functions from Seurat^62^ and Signac^63^.

### Preprocessing of C^me^CUT&Tag-BS/EM

Bisulfite-converted data were processed using an adapted WGBS pipeline from snakePipes. In brief, sequence alignment was performed using bwameth2, which relies on the bwa-mem2 as the underlying aligner^64^. The BAM files were converted to BigWig files, and peak calling was performed as described above.

CpG dinucleotide methylation profiles in the peaks were extracted from the resulting BAM files using MethylDackel^65^, with the following parameters: --mergeContext, --maxVariantFrac 0.1, --minDepth 5.

### Estimation of bisulfite/enzymatic conversion rate

Bisulfite treatment converts unmethylated cytosines (C) to uracil (U), which are read as thymine (T) during sequencing. The bisulfite conversion rate (CR) can be calculated as:

1 $${CR}=\frac{{{\rm{Converted}}}\; {{\rm{C}}}^{\prime} {{\rm{s}}}}{{{\rm{Converted}}}\; {{\rm{C}}}^{\prime} {{\rm{s}}}+{{\rm{Unconverted}}}\; {{\rm{C}}}^{\prime} {{\rm{s}}}}$$

Because DNA methylation predominantly occurs at CpG sites, cytosines in non-CpG (CHH) contexts are generally assumed to be unmethylated. Therefore, the bisulfite conversion rate is commonly estimated using CHH sites as follows:

2 $${CR}=\frac{{{\rm{Number}}}\; {{\rm{of}}}\; {{\rm{converted}}}\; {{\rm{C}}}^{\prime} {{\rm{s}}}\; {{\rm{in}}}\; {{\rm{CHH}}}}{{{\rm{Total}}}\; {{\rm{number}}}\; {{\rm{of}}}{{{\rm{C}}}}^{{\prime} }{{\rm{s}}}\; {{\rm{in}}}\; {{\rm{CHH}}}}$$

Two independent approaches were used for CR methylation on the genome CHH and the unmethylated bacteriophage T7 spike-in. CHH methylation levels were extracted using MethylDackel mbias –CHH.

### EM-seq quantification of DNA methylation reduction upon inhibitor treatment

CpG dinucleotide methylation profiles (BismarkCoverage file format) generated from MethylDackel were read into R using methRead function from MethylKit^66^. The CpG dinucleotide methylation profile was filtered by lo.count = 5, lo.perc = 1, hi.perc = 99. The methylation per CpG was calculated using the percMethylation function.

### PCA of histone modification CUT&Tag and C^me^CUT&Tag

To assess whether the binding profiles were consistent between C^me^CUT&Tag and C^me^CUT&Tag-BS/EM, the multiBamSummary tool from deepTools was used to quantify signal intensities over C^me^CUT&Tag peak regions. BAM files from histone modification CUT&Tag, 2xMBD2-CUT&Tag, and 2xMBD2-CUT&Tag-BS/EM experiments were included. Principal component analysis (PCA) was then performed in Python using the resulting matrix, focusing on the first two components for visualization.

### Coverage requirement of C^me^CUT&Tag and WGBS

To estimate the number of reads required to achieve specific coverage thresholds over C^me^CUT&Tag peaks, BAM files from all C^me^CUT&Tag sequencing runs were merged to create two datasets: one for nuclei-derived samples with 101 million aligned, properly paired reads, and one for genomic DNA with 191 million reads (75 bp read length). Each dataset was then subsampled to 2 million, 5 million, 10 million, 20 million, 50 million, and 100 million reads, and median per-base coverage over peaks was computed with plotCoverage from deepTools.

To provide a reference for whole-genome sequencing (WGS) on the human genome (3.2 Gb), the number of 75-bp reads required to reach equivalent coverage levels was estimated using the formula:

3 $${{\rm{Required}}}\; {{\rm{reads}}}=\frac{\left({{\rm{Target}}}\; {{\rm{coverage}}}\right)\times 3.2\times {10}^{9}\,{{\rm{bp}}}}{75\,{{\rm{bp}}}}$$

### C^me^CUT&Tag enrichment on MethylationEPIC regions

DNA methylation data were obtained from the Illumina MethylationEPIC array for two iPSC samples (GSE158089), yielding 517,789 probes with beta values. For each probe, the average beta value across replicates was calculated. Probes were then merged into contiguous 2 kb regions using bedtools merge -d 2000, resulting in a total of 70,676 regions. The merged regions were sorted by average methylation level (beta value), and CUT&Tag signal heatmaps for H3K4me3 and C^me^CUT&Tag (2xMBD2) were generated over these regions using plotHeatmap from deepTools.

### Differentially enriched regions during brain organoid development

BAM files from C^me^CUT&Tag (2 replicates per time point) and C^me^CUT&Tag-BS (2 replicates per time point) across three developmental stages (iPSC, day 16, and day 210) were analyzed using the DiffBind package in R. Pairwise differential enrichment analyses were performed between each combination of time points. Regions with a false discovery rate (FDR) < 0.05 were considered significantly differentially enriched. Diffbind regions on the standard chromosome (chr1-22) were then annotated using ChromHMM chromatin state models. GO analysis of the nearest genes was conducted as described above.

### DNA methylation profiling in differential regions with C^me^CUT&Tag-BS

To assess DNA methylation levels within the differentially bound regions, BAM files from C^me^CUT&Tag-BS were merged by time point (2 replicates each). CpG methylation coverage files were processed using the methylKit package in R, with a minimum coverage threshold of 5 (minCov = 5). Methylation data were then summarized over the previously identified DiffBind regions. The percentage of DNA methylation (%DNAme) for each region was extracted using the percMethylation() function. Regions were classified into four categories: no_detection, low (0–20%), mid (20–80%), and high (80–100%) DNA methylation.

### crossNN prediction of tumor biopsies

C^me^CUT&Tag-BS/EM data were pre-processed as described above. CpG methylation calls (MethylDackel output in Bismark coverage format) were lifted over from hg38 to hg19 and intersected with Illumina EPIC 450k probe coordinates. Matched CpGs were converted to bedMethyl format for compatibility with the classifier. CpGs with sequencing coverage <5× were excluded. Samples with fewer than 300 EPIC-matched CpG features after filtering were removed from downstream analysis.

The resulting probe-level methylation profiles were used as input to the crossNN classifier pre-trained on 2801 EPIC 450k reference samples^3^.

To account for potential assay-specific bias introduced by C^me^CUT&Tag-BS/EM, class logits for tumor biopsies were calibrated by subtracting the mean logit vector derived from control brain organoids (averaged across samples). The calibrated logits were subsequently transformed into class probabilities using a softmax function. Predicted methylation class (MC) was defined as the class with the maximum posterior probability.

For the construction of the confusion matrix, predicted MC labels were aggregated to methylation class family (MCF) according to the classification scheme^3^.

### Reporting summary

Further information on research design is available in the Nature Portfolio Reporting Summary linked to this article.

## Supplementary information

Supplementary Information

Description of Additional Supplementary Files

Supplementary Data 1

Supplementary Data 2

Reporting Summary

Transparent Peer Review file

## Source data

Source Data

## Data Availability

The data supporting the findings of this study are available from the corresponding authors upon request. The data generated in this study have been deposited in the GEO database under accession code GSE320203. Source data for the figures and Supplementary Figs. are provided as a Source Data file. Previously published data used in this paper include: GSE25970. GSE82022. GSE150122. GSE158089. GSE159071. GSE16368. GSE203377. GSE179673. GSE35050. GSE70847. For details of the publicly available datasets analyzed in this study, please refer to the “Methods” section. Source data are provided with this paper.

## Code availability

All generated code is available on GitHub: https://github.com/EpiGN-EPFL/CmeCUT-Tag and on Zenodo: 10.5281/zenodo.19555724.
