# Supplementary material for: A scalable Tn5-based method for genome-wide DNA methylation profiling in development and disease
Source: Nat Commun. 2026 May 22;17:6736. doi: 10.1038/s41467-026-73325-4 (PMC13385352; doi:10.1038/s41467-026-73325-4)
Supplement: Supplementary file 1 — Supplementary Information [file 41467_2026_73325_MOESM1_ESM.pdf]

Supplementary Materials for

A scalable Tn5-based method for genome-wide DNA methylation profiling in development and disease

Hanrong Hu[1]*, Nahuel Simonet[1]*, Ece Naz Bilgiç[1], Heather Murray[1], Regina Reimann[2], Markus Rechsteiner[2], Fides Zenk[1]✉
*These authors contributed equally to the work
✉Correspondence: fides.zenk@epfl.ch

[1] Ecole Polytechnique Federale de Lausanne (EPFL), School of Life Sciences, Brain Mind Institute, EpiGN – NeuroNA Chair in Epigenomics of Neurodevelopmental Disorders, Station 19, 1015 Lausanne, Switzerland, Chemin des Mines 9, 1202 Geneve, Switzerland
[2] UniversitätsSpital Zürich, Schmelzbergstrasse 12, 8091 Zürich, Switzerland

Table of contents

Supplementary Figure 1: Example workflow of C^{me}-CUT&Tag on nuclei
Supplementary Figure 2: Characterization of MBD-binding profiles *in-situ* in nuclei.
Supplementary Figure 3: C^{me}CUT&Tag shows high efficiency for low cell numbers and low genomic DNA input.
Supplementary Figure 4: Methylation profiles obtained with C^{me}CUT&Tag are similar to MeDIP and MBD-Seq.
Supplementary Figure 5: C^{me}CUT&Tag is specific to DNA methylation.
Supplementary Figure 6: C^{me}CUT&Tag-BS and C^{me}CUT&Tag-EM preserve the binding profile of C^{me}CUT&Tag and reach base-pair resolution.
Supplementary Figure 7: C^{me}CUT&Tag(-BS/EM) tracks the DNA methylation dynamics of brain organoid development.
Supplementary Figure 8: C^{me}CUT&Tag profiles DNA methylation in zebrafish.

Supplementary Fig. 1

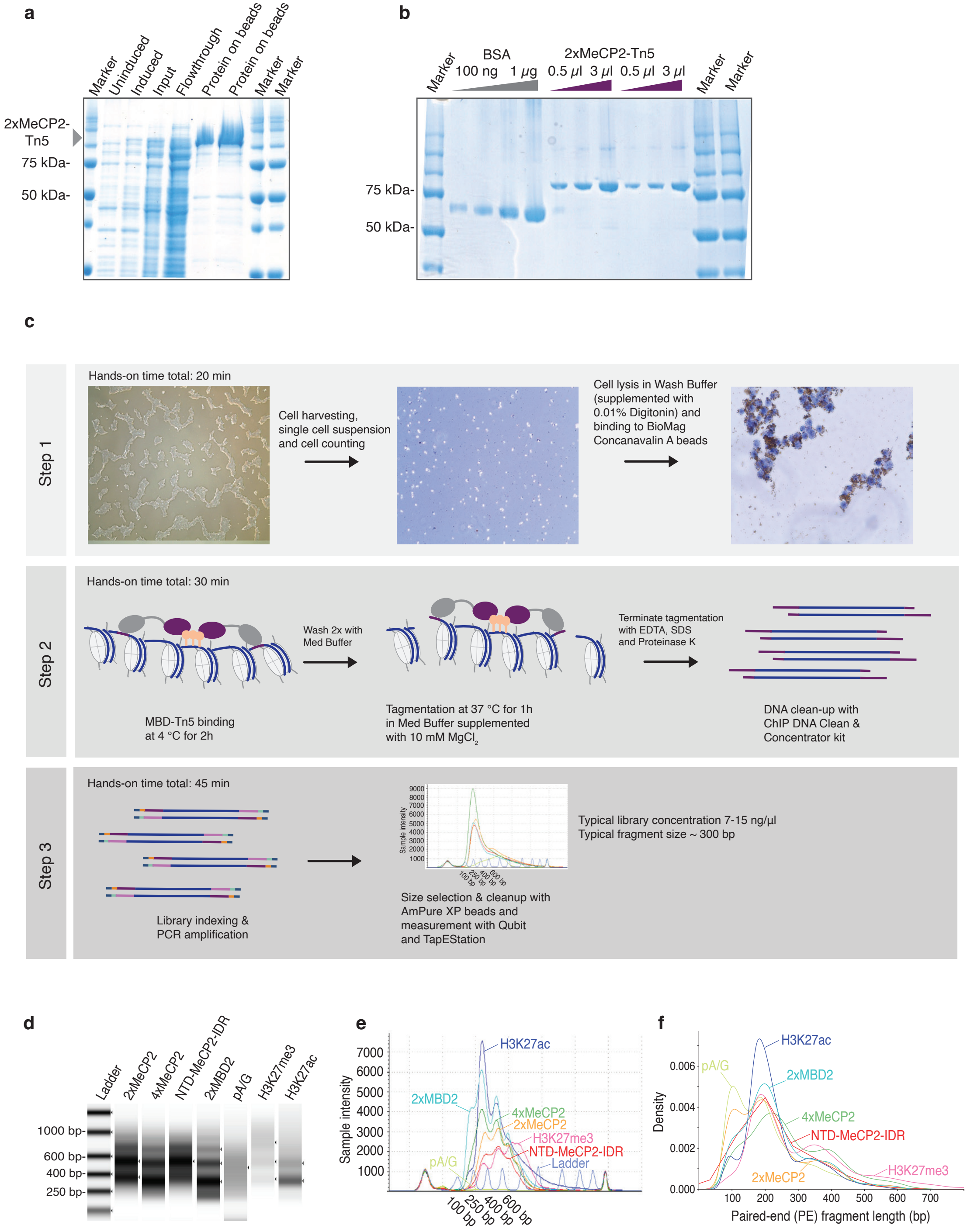

Supplementary Figure 1. **Example workflow of C^{me}CUT&Tag on nuclei.**

a, Representative uncropped gel images of the protein purification. The arrowhead indicates the position of 2xMeCP2-Tn5 before cleavage of the Chitin-binding-domain tag.

b, Gel images of purified and active 2xMeCP2-Tn5 next to a BSA-standard.

c, Example workflow showing representative images of iPSCs and nuclei preparations.

d, Tapestation-generated gel-like images of representative libraries prepared by CUT&Tag and C^{me}CUT&Tag on iPSC nuclei.

e, Tapestation profiles of representative libraries prepared by CUT&Tag and C^{me}CUT&Tag on iPSC nuclei.

f, Paired-end BAM fragment length distribution from aligned BAM files.

Supplementary Fig. 2

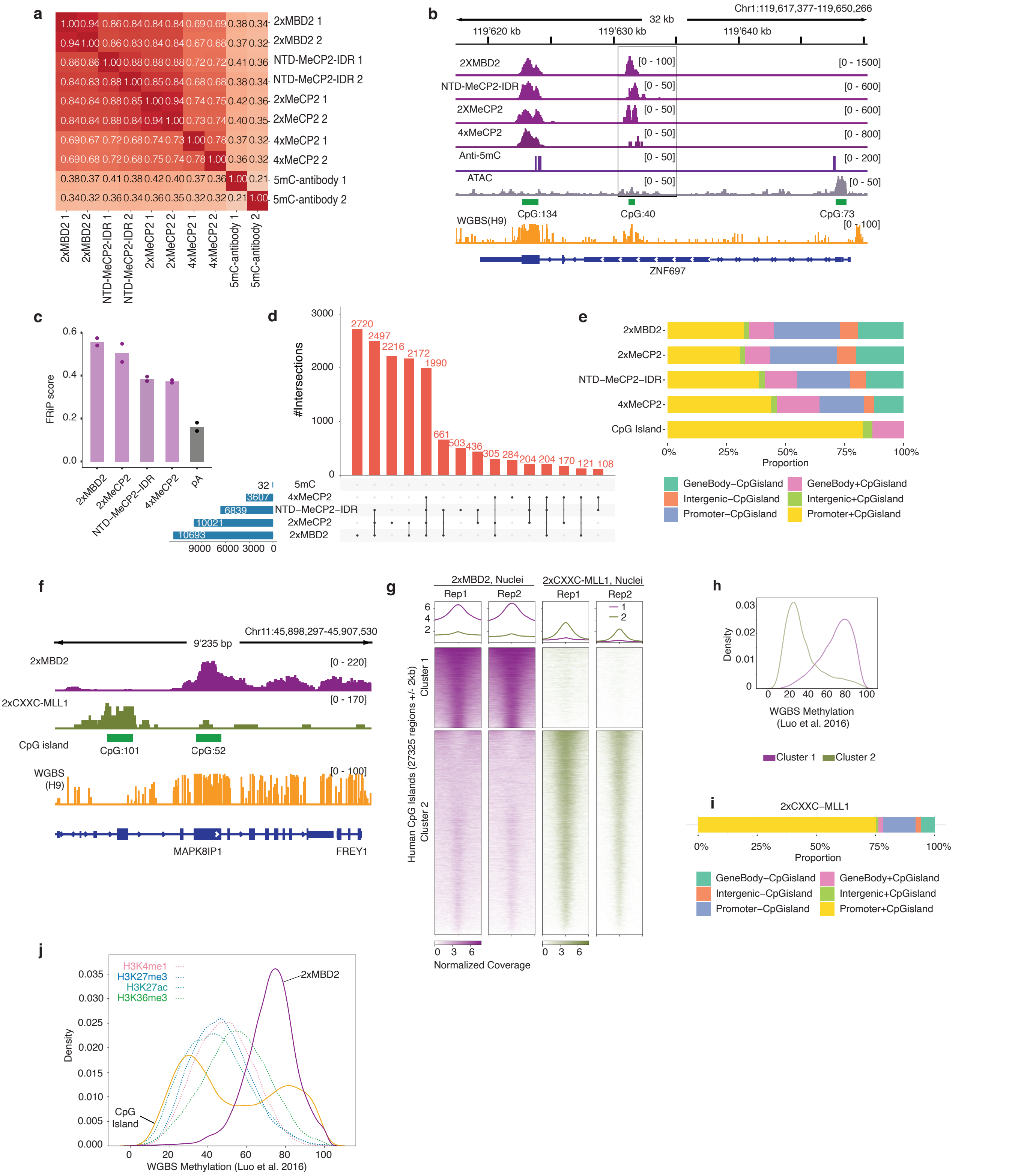

Supplementary Figure 2. **Characterization of MBD-binding profiles *in-situ* in nuclei.**

a, Pearson correlation of MBD-constructs used in C^{me}CUT&Tag and 5mC antibody (2 replicates each) used in CUT&Tag on BAM files on all C^{me}CUT&Tag peaks.

b, Different MBD constructs and 5mC-antibody binding to CpG-rich genomic regions at the *ZNF697* locus on iPSC nuclei, along with WGBS data on hESCs (H9)[18]. One representative replicate of each construct is shown.

c, Fraction of fragments in peaks (FRiP scores) of MBD-constructs compared to an untargeted Tn5 fused to protein A (pA), indicating superior performance of 2xMBD2 (see Supplementary Data 1 for additional information on all samples). Bars show the mean of 2 replicates.

d, UPSET plot of each MBD-construct's peaks (consensus of two replicates) showing strong overlap between all constructs. The blue barplot shows the total number of peaks for each construct.

e, ChIPseeker analysis of each MBD-construct peak set. Source data are provided as a Source Data file.

f, Genome Browser Snapshot of 2xMBD2 and 2xCXXC-MLL1 identifies highly and lowly methylated CpG islands, respectively (n = 2, showing one representative replicate each) along with WGBS data on hESCs (H9)[18].

g, Heatmap of 2xMBD2 and 2xCXXC-MLL1 CUT&Tag (n = 2, log2 normalized) on human CpG islands (standard chromosomes). K-means clustering (k = 2) analysis reveals two distinct clusters, where CpG islands in cluster 1 are enriched in 2xMBD2 signals, while the ones in cluster 2 are enriched in 2xCXXC-MLL1.

h, Density plot showing the CpG methylation in the clusters of g., confirming that MBD-bound CpG islands show high DNA methylation levels while CXXC-bound CpG islands show low DNA methylation levels.

i, ChIPseeker analysis of 2xCXXC-MLL1 peaks. Source data are provided as a Source Data file.

j, Density plot showing the distribution of average DNA methylation levels (% mCpG, from WGBS) across peaks identified by 2xMBD2 C^{me}CUT&Tag and histone modification CUT&Tag. C^{me}CUT&Tag peaks are predominantly located in regions with >40% DNA methylation, highlighting its specificity for highly methylated loci.

Supplementary Fig. 3

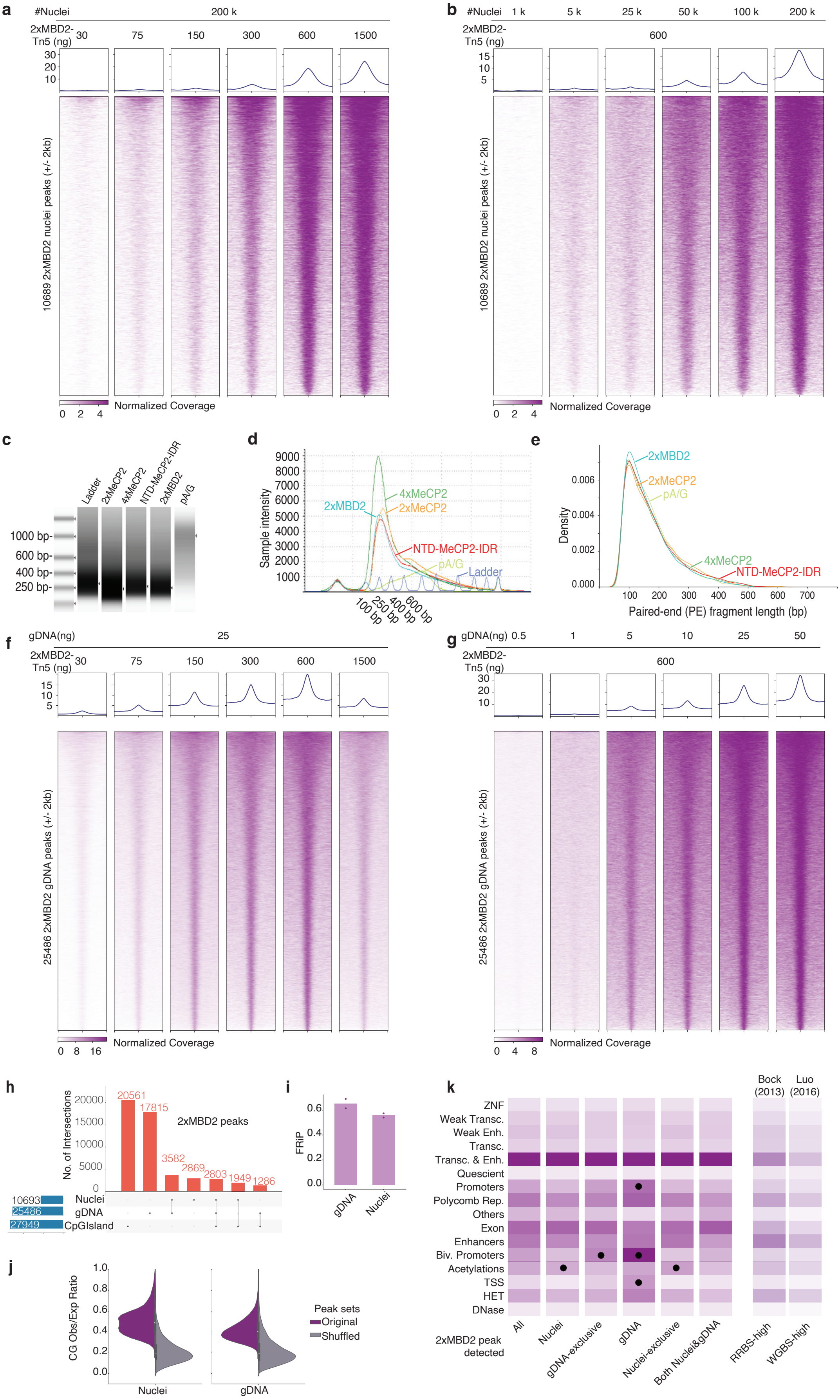

Supplementary Figure 3. **C^{me}CUT&Tag shows high efficiency for low cell numbers and low genomic DNA input.**

a, Titration of input 2xMBD2-Tn5 fusion protein amount (30 ng – 1500 ng) on 200 k input nuclei (iPSCs). Heatmaps include one representative replicate of each condition (spike-in normalized).

b, Serial dilution of input nuclei (1k – 200k iPSCs) with 600 ng 2xMBD2-Tn5 fusion protein. Heatmaps include one representative replicate of each condition (spike-in normalized).

c, Tapestation-generated gel-like images of representative libraries prepared by CUT&Tag and C^{me}CUT&Tag on iPSC genomic DNA.

d, Tapestation profiles of representative libraries prepared by CUT&Tag and C^{me}CUT&Tag on iPSC genomic DNA.

e, Paired-end bam fragment length distribution from aligned BAM files of C^{me}CUT&Tag on genomic DNA.

f, Titration of input 2xMBD2-Tn5 fusion protein amount (30 ng – 1500 ng) on 25 ng input genomic DNA. Heatmaps include one representative replicate of each condition (spike-in normalized).

g, Serial dilution of input genomic DNA (0.5 ng – 50 ng) with 600 ng 2xMBD2-Tn5 fusion protein. Heatmaps include one representative replicate of each condition (spike-in normalized).

h, UPSET plot of CpG island capture of 2xMBD2-CUT&Tag on genomic DNA and nuclei. More than 50% of the peaks detected in nuclei are also detected in isolated DNA. Blue barplot shows the total number of peaks for each construct.

i, FRiP scores of 2xMBD2-CUT&Tag on genomic DNA and nuclei. Bars show the mean of 2 replicates.

j, CpG enrichment ratios (observed/expected) of nuclei- and gDNA-derived peak sets are higher than those of their respective shuffled controls.

k, ChromHMM models of 2xMBD2-CUT&Tag peak intersection with high methylation (>80% mCpG) regions from RRBS (hES cell line H9)[5] and WGBS (hiPSC)[18] datasets, showing that C^{me}CUT&Tag on gDNA and nuclei generally recover very similar regions, with slightly higher enrichment of gDNA for bivalent promoters. The category all contains the total of all peaks detected in isolated DNA and nuclei, gDNA contains all peaks detected in isolated DNA, nuclei contains all peaks detected in nuclei, gDNA exclusive contains peaks that are uniquely detected in isolated DNA, and nuclei exclusive contains peaks that are uniquely detected in nuclei. The category both nuclei and gDNA contains the overlapping peak set. Source data are provided as a Source Data file.

Supplementary Fig. 4

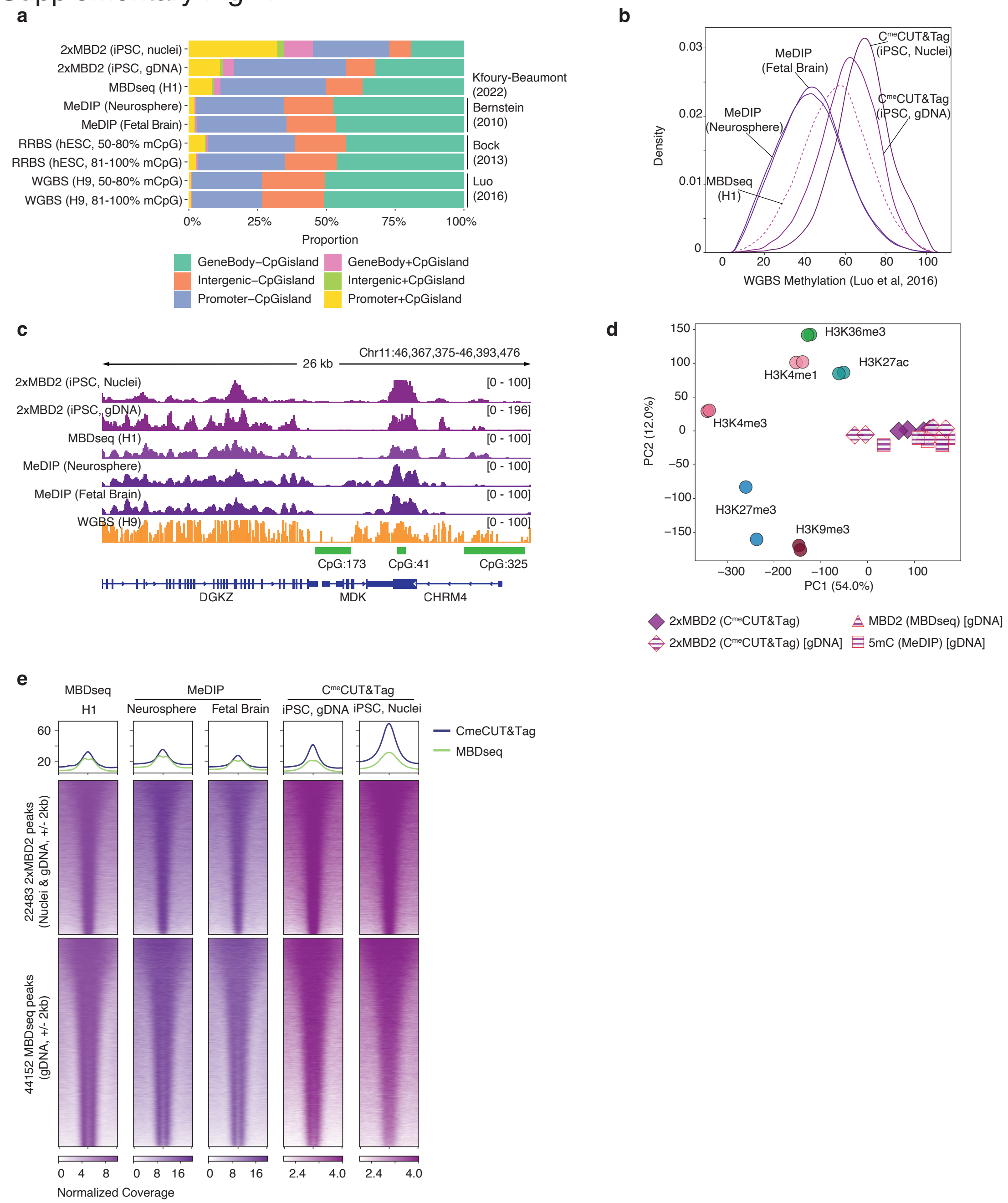

Supplementary Figure 4. **Methylation profiles obtained with C^{me}CUT&Tag are similar to MeDIP and MBD-Seq.**

a, ChIPseeker analysis of peak sets from 2xMBD2 in iPSC nuclei and gDNA, enrichment-based methods (MeDIP on fetal brain and cortex-derived neurospheres, and MBD-seq on hESC), and bisulfite sequencing (WGBS and RRBS on hESC). C^{me}CUT&Tag captures genomic regions comparable to those identified by existing approaches, especially in gDNA.

b, Density plot comparing average DNA methylation (% mCpG, H9 WGBS) across peak sets from 2xMBD2 C^{me}CUT&Tag (nuclei and gDNA), MBD-seq (H1 cells), and MeDIP (fetal brain and cortex-derived neurospheres), showing higher sensitivity of C^{me}CUT&Tag to regions with high DNA methylation.

c, Example tracks comparing enrichment-based DNA methylation profiling methods at CpG-rich regions, together with H9 WGBS data[18].

d, PCA of C^{me}CUT&Tag (nuclei and gDNA), MeDIP, MBD-seq, and histone modification CUT&Tag signals (log2 transformed) computed over C^{me}CUT&Tag peaks.

e, Heatmap showing averaged 2xMBD2 C^{me}CUT&Tag signals (gDNA and nuclei, two replicates each) alongside MBD-seq (n = 4) and MeDIP signals (n = 2) at their respective peak regions. BigWig signals are averaged among replicates.

Supplementary Fig. 5

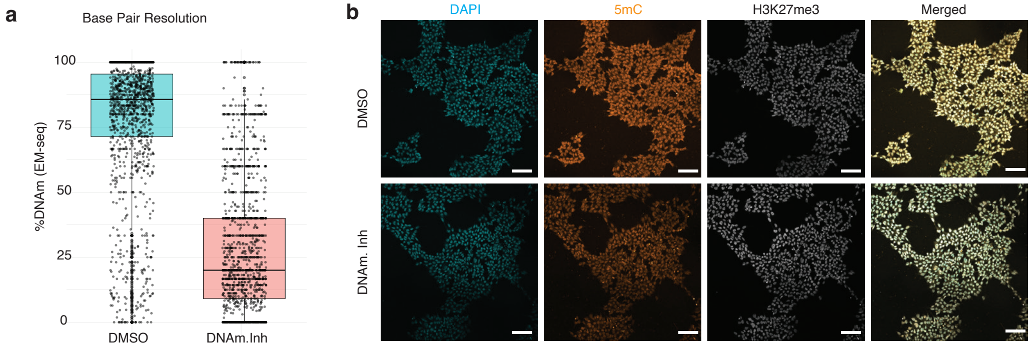

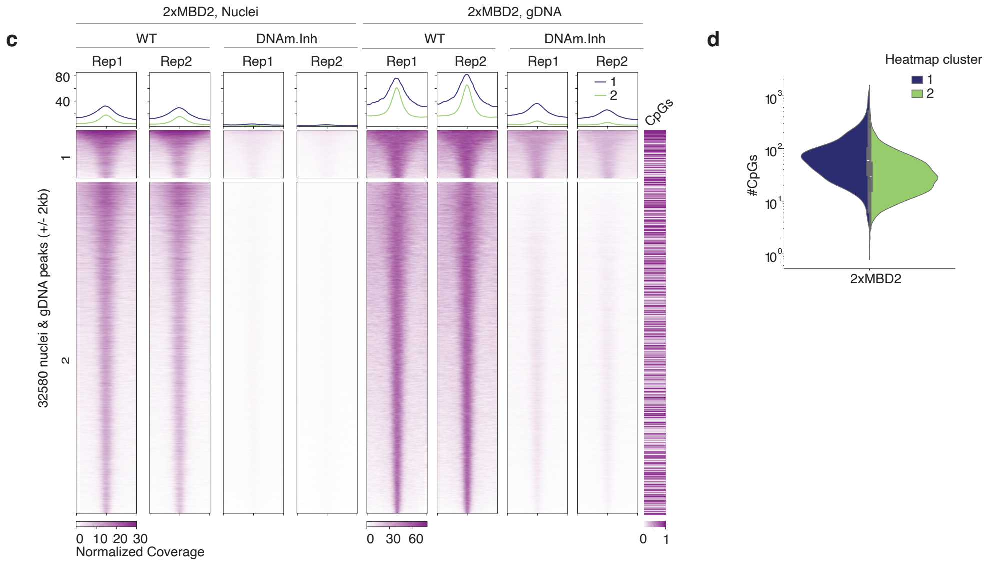

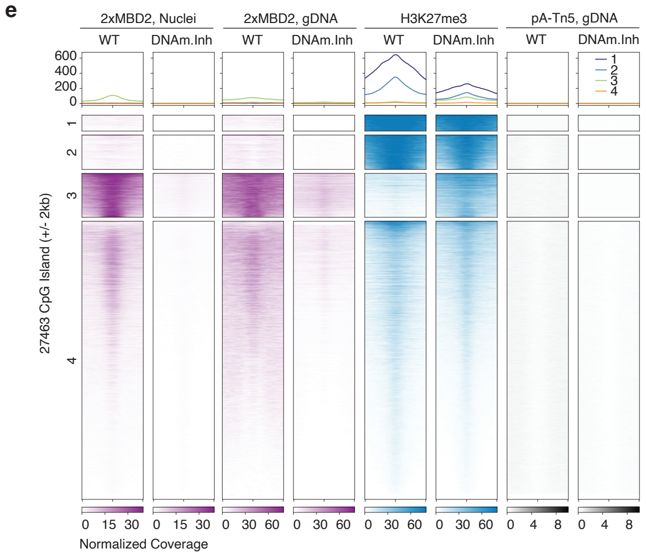

f

Gene Ontology Enrichment Analysis

Gain of H3K27me3 (DNAm.Inh vs WT):

Homophilic cell adhesion via plasma membrane adhesion molecules (3.72e-09)
Regulation of membrane potential (2.73e-04)
Inorganic cation import across plasma membrane (1.84e-03)
Import across plasma membrane (3.55e-03)
Cellular component assembly involved in morphogenesis (5.29e-03)

Loss of H3K27me3 (DNAm.Inh vs WT):

Axonogenesis (1.03e-13)
Regulation of neuron projection development (1.40e-11)
Learning or memory (1.40e-11)
Regulation of synapse structure or activity (9.52e-09)
Regulation of synapse organization (3.03e-08)

Supplementary Figure 5. **C^{me}CUT&Tag is specific to DNA methylation.**

a, EM-seq quantification of the DNA methylation at base-pair resolution (1308 regions) shows that DNMT1 inhibitor treatment reduced the DNAm% from 80% to 20%. The center line denotes the median; boxes denote lower and upper quartiles (Q1 and Q3, respectively); whiskers denote 1.5× the interquartile region below Q1 and above Q3; and points denote outliers.

b, Immunofluorescence staining of the DNMT1 inhibitor-treated cells (n = 2, showing one representative replicate) shows the decrease of DNA methylation (5mC staining, in yellow) and the increase of H3K27me3 (in grey). Scale bar, 100 µm.

c, Heatmap of C^{me}CUT&Tag signals at C^{me}CUT&Tag peak regions in nuclei and genomic DNA before and after DNMT1 inhibitor treatment (n = 2), showing widespread signal loss and a subset of peaks with residual enrichment.

d, Violin-Plot showing CpG content analysis of the clusters in c. Peaks retaining residual signal are characterized by high CpG counts.

e, Heatmaps showing loss of 2xMBD2 binding at CpG islands in DNMT1-inhibitor-treated human iPSCs (two replicates averaged). Clustering reveals region-specific gain or loss of H3K27me3 upon DNA methylation depletion.

f, Gene ontology analysis of differentially enriched H3K27me3 peaks (Fig. 2d, FDR < 0.05). Source data are provided as a Source Data file.

Supplementary Fig. 6

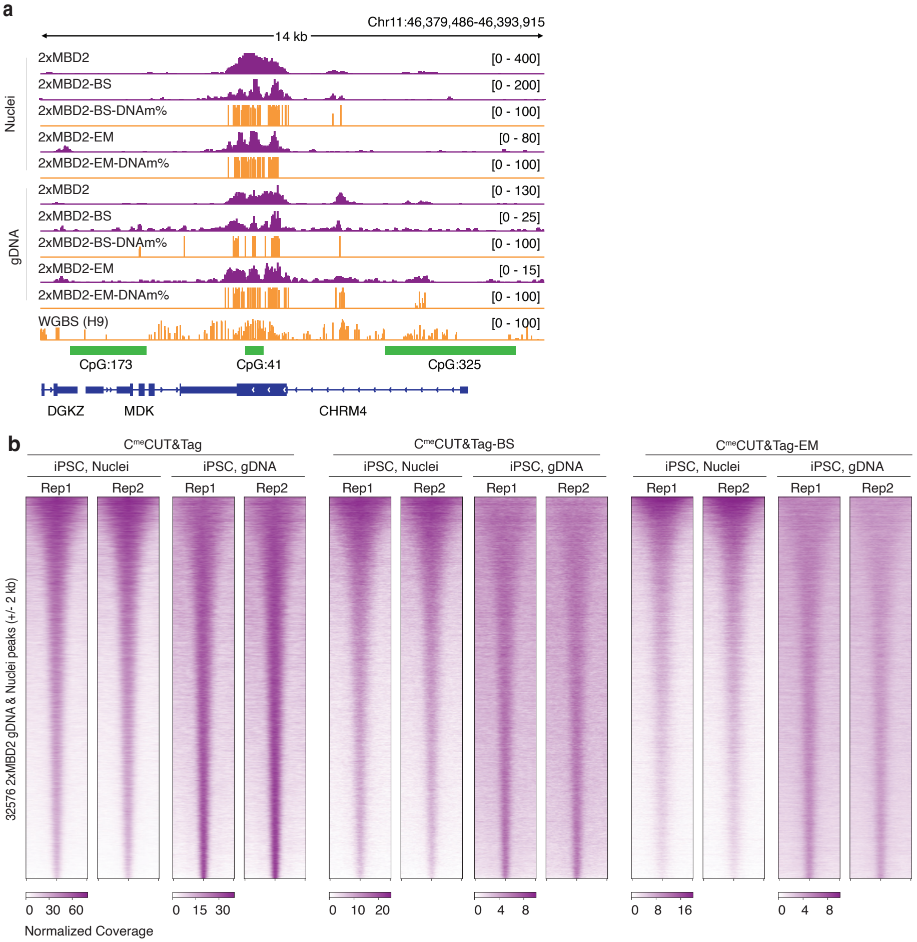

Supplementary Figure 6. **C^{me}CUT&Tag-BS and C^{me}CUT&Tag-EM preserve the binding profile of C^{me}CUT&Tag and reach base-pair resolution.**

a, IGV snapshot of 2xMBD2-C^{me}CUT&Tag on genomic DNA and nuclei from iPSCs, before and after bisulfite or enzymatic conversion, compared to published WGBS from H9 cells[18].

b, Heatmaps of 2xMBD2-C^{me}CUT&Tag on genomic DNA and nuclei, before and after bisulfite or enzymatic conversion on the total peaks of gDNA and nuclei (n = 2).

Supplementary Fig. 7

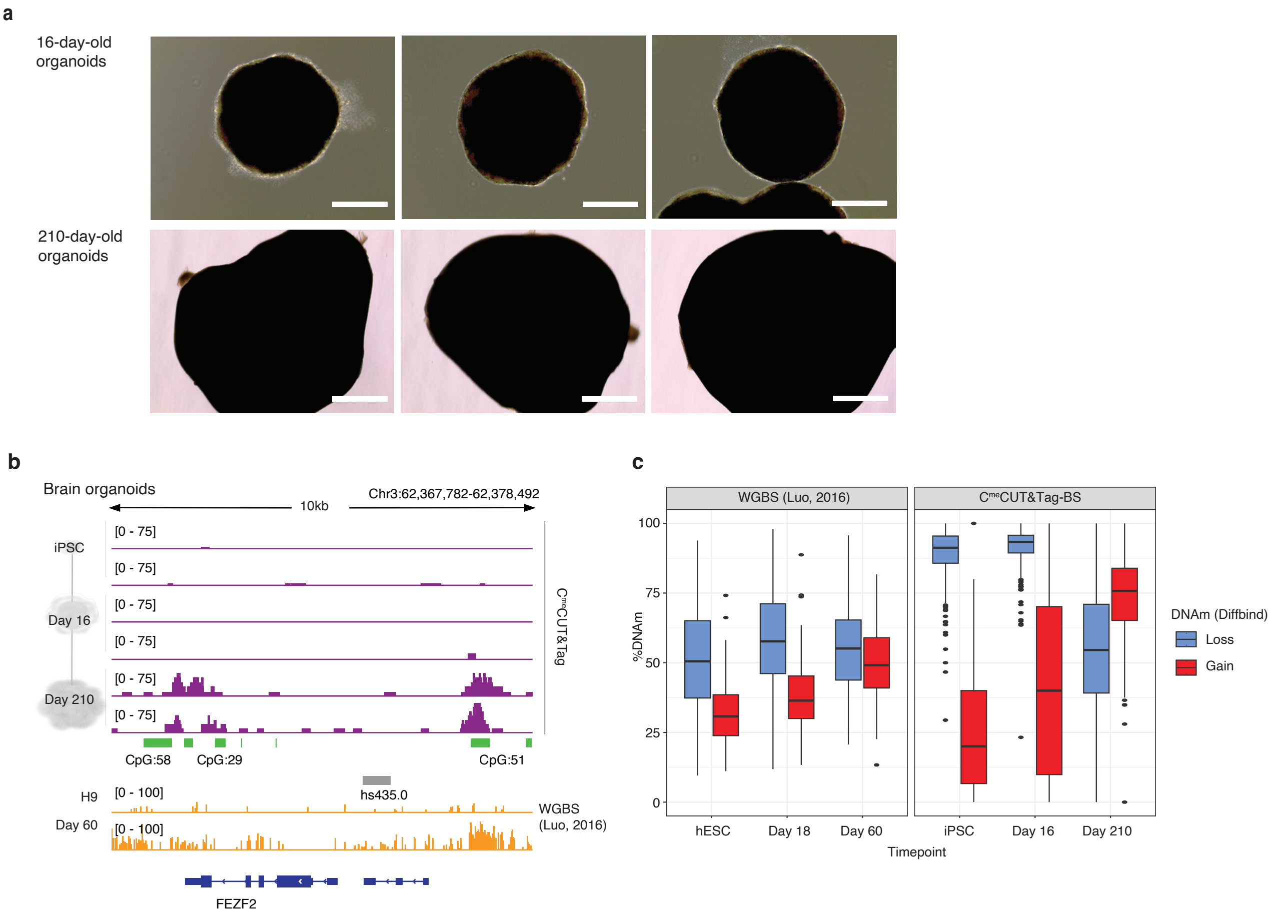

Supplementary Figure 7. **C^{me}CUT&Tag(-BS/EM) tracks the DNA methylation dynamics of brain organoid development.**

a, Images of the organoids used in the analysis. Scale bar, 1000 µm.

b, Example of C^{me}CUT&Tag profiles at different organoid ages on the *FEZF2* locus, along with the WGBS profiling of hESC (H9) and brain organoid at day 60[18] (n = 2).

c, Box-plot characterizing the dynamic behavior of DNA-methylation at the differential peak set from Figure 4e quantified by WGBS[18] (left) and C^{me}CUT&Tag-BS (right) across brain organoid development. The center line denotes the median; boxes denote lower and upper quartiles (Q1 and Q3, respectively); whiskers denote 1.5× the interquartile region below Q1 and above Q3; and points denote outliers. Source data are provided as a Source Data file.

Supplementary Fig. 8

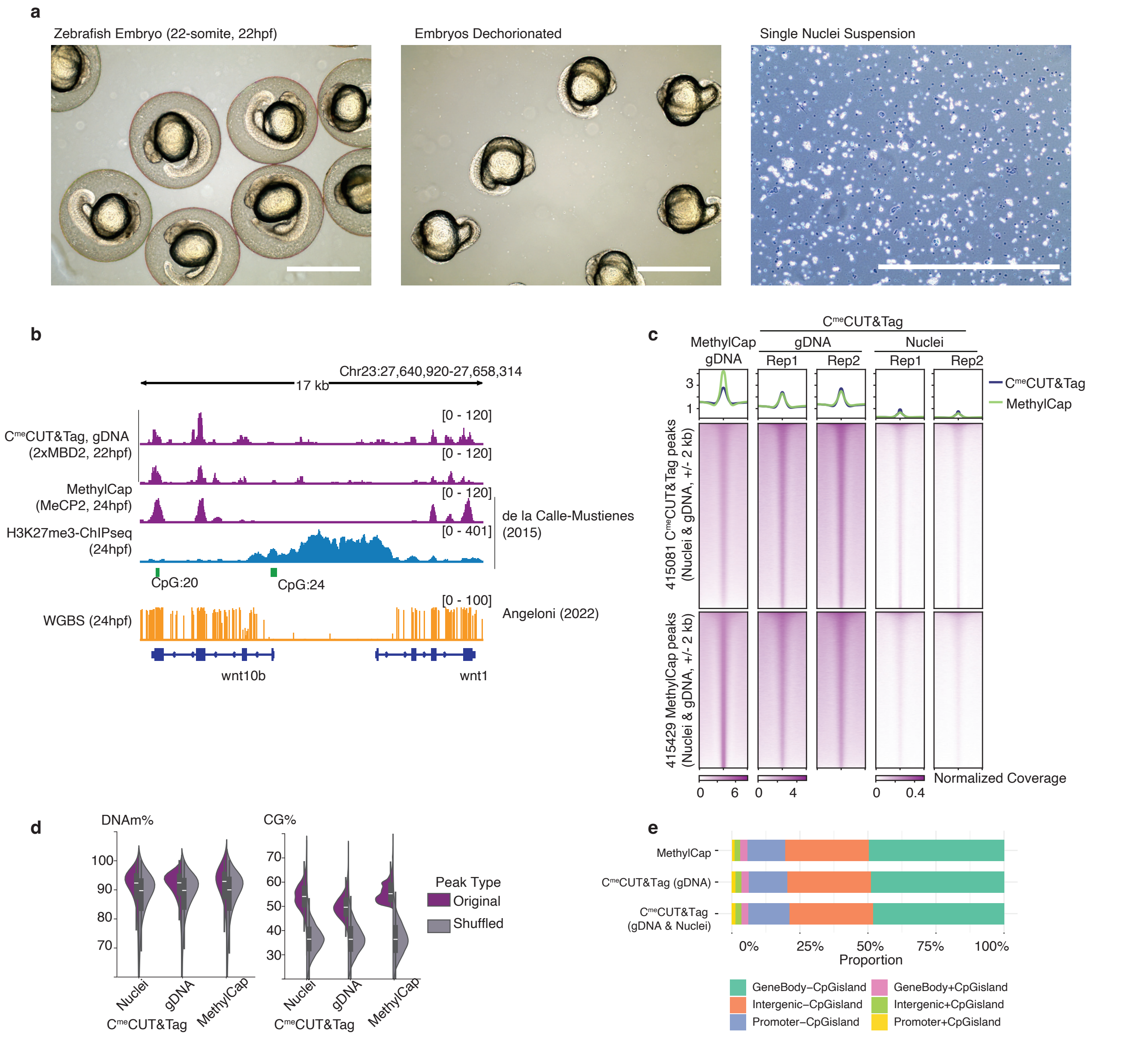

Supplementary Figure 8. **C^{me}CUT&Tag profiles DNA methylation in zebrafish.**

a, Representative images of zebrafish embryos at around 22 hours post fertilization (hpf) used in the analysis. Scale bar, 1000 μm.

b, Example of the BigWig tracks of C^{me}CUT&Tag (gDNA) and published MethylCap on *WNT1* locus (n = 2), along with published H3K27me3 ChIP-seq data[32] and whole genome methylation data[33].

c, Heatmap showing averaged 2xMBD2 C^{me}CUT&Tag signals (log2 transformed) alongside published MethylCap signals at their respective peak regions (consensus of available replicates, n = 2 for C^{me}CUT&Tag, n = 1 for MethylCap) in zebrafish[32].

d, Methylation percentage (DNAm% measured from published genome methylation data[33]) and number of CpGs analysis of C^{me}CUT&Tag and MethylCap peaks[32]. The center line denotes the median; boxes denote lower and upper quartiles (Q1 and Q3, respectively); whiskers denote 1.5× the interquartile region below Q1 and above Q3; and points denote outliers.

e, ChIPSeeker analysis of C^{me}CUT&Tag and published MethylCap peaks[32]. Source data are provided as a Source Data file.
