# Supplementary material for: A scalable Tn5-based method for genome-wide DNA methylation profiling in development and disease
Source: Nat Commun. 2026 May 22;17:6736. doi: 10.1038/s41467-026-73325-4 (PMC13385352; doi:10.1038/s41467-026-73325-4)
Supplement: Supplementary file 2 — Description of Additional Supplementary Files [file 41467_2026_73325_MOESM2_ESM.pdf]

Description of Additional supplementary files

Supplementary Data 1: Additional information on all samples used in this study.

Supplementary Data 2: Comparison of sequencing-based DNA methylation profiling methods.
