# Supplementary material for: A scalable Tn5-based method for genome-wide DNA methylation profiling in development and disease
Source: Nat Commun. 2026 May 22;17:6736. doi: 10.1038/s41467-026-73325-4 (PMC13385352; doi:10.1038/s41467-026-73325-4)
Supplement: Supplementary file 5 — Reporting Summary [file 41467_2026_73325_MOESM5_ESM.pdf]

nature portfolio

Corresponding author(s): Fides Zenk

Last updated by author(s): 2026.02.25

Reporting Summary

Nature Portfolio wishes to improve the reproducibility of the work that we publish. This form provides structure for consistency and transparency in reporting. For further information on Nature Portfolio policies, see our Editorial Policies and the Editorial Policy Checklist.

Statistics

For all statistical analyses, confirm that the following items are present in the figure legend, table legend, main text, or Methods section.

n/a	Confirmed	
☐	☒	The exact sample size (*n*) for each experimental group/condition, given as a discrete number and unit of measurement
☐	☒	A statement on whether measurements were taken from distinct samples or whether the same sample was measured repeatedly
☐	☒	The statistical test(s) used AND whether they are one- or two-sided<br>*Only common tests should be described solely by name; describe more complex techniques in the Methods section.*
☒	☐	A description of all covariates tested
☐	☒	A description of any assumptions or corrections, such as tests of normality and adjustment for multiple comparisons
☐	☒	A full description of the statistical parameters including central tendency (e.g. means) or other basic estimates (e.g. regression coefficient) AND variation (e.g. standard deviation) or associated estimates of uncertainty (e.g. confidence intervals)
☐	☒	For null hypothesis testing, the test statistic (e.g. *F*, *t*, *r*) with confidence intervals, effect sizes, degrees of freedom and *P* value noted<br>*Give P values as exact values whenever suitable.*
☒	☐	For Bayesian analysis, information on the choice of priors and Markov chain Monte Carlo settings
☒	☐	For hierarchical and complex designs, identification of the appropriate level for tests and full reporting of outcomes
☐	☒	Estimates of effect sizes (e.g. Cohen's *d*, Pearson's *r*), indicating how they were calculated

Our web collection on statistics for biologists contains articles on many of the points above.

Software and code

Policy information about availability of computer code

Data collection

Basecalls were performed using bases2fastq version 1.8.0. Nextera adapters were trimmed with minimal read length tolerated post-trimming: 35 bp.

Data analysis

All packages used are thoroughly described in the methods section.
CUT&Tag sequencing data was processed using an adapted DNAmapping pipeline from snakePipes (v3.1.0). CUT&Tag-BS/EM data were processed using an adapted WGBS pipeline from snakePipes. Broad peaks were called from the BAM files of each sequencing run using MACS3 (v3.0.1) with the parameters --broad-cutoff 0.1, --nolambda.
deepTools (v3.5) was used for comparing the correlation of BigWigs/BAM files.Customized code was used for visualization in Python (pandas, numpy, matplotlib, seaborn).
MethylDackel (v 0.5.1) was used for CpG methylation calling. Differential enrichment analysis was done using Diffbind and methlyKit in R (v4.4). For crossNN prediction of the brain tumor biopsy, CpG methylation calls (MethylDackel output in Bismark coverage format) were lifted over from hg38 to hg19 and intersected with Illumina EPIC 450k probe coordinates.
For single-cell CmeCUT&Tag, Seurat (v5.4.0) and Signac(v1.16.0) in R (v4.5) were used.
We have deposited the code used to the github repository (https://github.com/EpiGN-EPFL/CmeCUT-Tag) and on Zenodo (https://doi.org/10.5281/zenodo.19555724)

For manuscripts utilizing custom algorithms or software that are central to the research but not yet described in published literature, software must be made available to editors and reviewers. We strongly encourage code deposition in a community repository (e.g. GitHub). See the Nature Portfolio guidelines for submitting code & software for further information.

Data

Policy information about availability of data

All manuscripts must include a data availability statement. This statement should provide the following information, where applicable:

- Accession codes, unique identifiers, or web links for publicly available datasets
- A description of any restrictions on data availability
- For clinical datasets or third party data, please ensure that the statement adheres to our policy

All data is available from our GEO account: https://www.ncbi.nlm.nih.gov/geo/query/acc.cgi?acc=GSE320203
Thes are previously published dataset:
GSE25970 [https://www.ncbi.nlm.nih.gov/geo/query/acc.cgi?acc=GSE25970]
GSE82022 [https://www.ncbi.nlm.nih.gov/geo/query/acc.cgi?acc=GSE82022]
GSE150122 [https://www.ncbi.nlm.nih.gov/geo/query/acc.cgi?acc= GSE150122]
GSE158089 [https://www.ncbi.nlm.nih.gov/geo/query/acc.cgi?acc=GSE158089]
GSE159071 [https://www.ncbi.nlm.nih.gov/geo/query/acc.cgi?acc=GSE159071]
GSE16368 [https://www.ncbi.nlm.nih.gov/geo/query/acc.cgi?acc=GSE16368]
GSE203377 [https://www.ncbi.nlm.nih.gov/geo/query/acc.cgi?acc=GSE203377]
GSE179673 [https://www.ncbi.nlm.nih.gov/geo/query/acc.cgi?acc=GSE179673]
GSE35050 [https://www.ncbi.nlm.nih.gov/geo/query/acc.cgi?acc=GSE35050]
GSE70847 [https://www.ncbi.nlm.nih.gov/geo/query/acc.cgi?acc=GSE70847]

Research involving human participants, their data, or biological material

Policy information about studies with human participants or human data. See also policy information about sex, gender (identity/presentation), and sexual orientation and race, ethnicity and racism.

Reporting on sex and gender	We have not collected information about sex and gender.
Reporting on race, ethnicity, or other socially relevant groupings	We have not collected or used information about race, ethnicity or other groupings.
Population characteristics	not applicable
Recruitment	Human material was collected fully anonymized and after informed consent.
Ethics oversight	The use of human ES cells for the generation of brain organoids was approved by the Commission cantonale d'éthique de la recherche (CCER) (2024-00680) and the Swiss federal office of public health.<br>Under the Swiss Human Research Act, research performed with fully anonymized human specimens does not require an institutional review for research as long as consent was approved in the first place. The human derived iPCS and cancer cell lines used in the study are available commercially and usage is regulated within the MTA.

Note that full information on the approval of the study protocol must also be provided in the manuscript.

Field-specific reporting

Please select the one below that is the best fit for your research. If you are not sure, read the appropriate sections before making your selection.

☒ Life sciences ☐ Behavioural & social sciences ☐ Ecological, evolutionary & environmental sciences

For a reference copy of the document with all sections, see nature.com/documents/nr-reporting-summary-flat.pdf

Life sciences study design

All studies must disclose on these points even when the disclosure is negative.

Sample size	We used several iPSC collections/embryoid bodies/organoids (per experiment) from different cell lines. No statistical methods were used to pre-determine sample sizes, but our sample sizes are similar to those reported in previous publications.
Data exclusions	No dataset were excluded from the study. For single cell analysis we performed strict quality filtering and exclduded individual cells that did contain high quality data. For the details and filtering criteria see: Methods
Replication	All Cut&Tag based experiments were performed in biological duplicates. All attempts of replication were successful.
Randomization	All biological samples used were randomly chosen from a larger sample pool.

Blinding	First computational analysis and inspection of the data were performed blinded.

Reporting for specific materials, systems and methods

We require information from authors about some types of materials, experimental systems and methods used in many studies. Here, indicate whether each material, system or method listed is relevant to your study. If you are not sure if a list item applies to your research, read the appropriate section before selecting a response.

Materials & experimental systems

n/a	Involved in the study	
☐	☒	Antibodies
☐	☒	Eukaryotic cell lines
☒	☐	Palaeontology and archaeology
☐	☒	Animals and other organisms
☒	☐	Clinical data
☒	☐	Dual use research of concern
☒	☐	Plants

Methods

n/a	Involved in the study	
☒	☐	ChIP-seq
☒	☐	Flow cytometry
☒	☐	MRI-based neuroimaging

Antibodies

Antibodies used	5mC Diagenode C1520003, RD-007<br>H3K27ac Diagenode C15410196, A1723-0041D<br>H3K27me3 Diagenode C15410195, A0824D<br>H3K36me3 Abcam AB9050, 1063779-1<br>H3K4me1 Diagenode C15410194, A1862D<br>H3K4me3 Diagenode C15410003, A1052D<br>H3K9me3 Abcam ab176916, GR3218257-2<br>Secondary anti mouse 488 (Thermo, A21202)<br>Secondary anti rabbit 488 (Thermo, A10040)<br>For CUT&Tag 1ug were used per experiment per antibody. For IF stainings H3K27me3 was diluted 1:1000 and 5mC was diluted to 1:5000. Secondary antibodies were diluted 1:500.
Validation	Antibodies used in this study are commercially available and have been validated by the manufacturer. We further validated antibodies against H3K27me3, H3K27ac by Western Blot in the control and upon inhibition of the respective epigenetic writer.<br>H3K27me3 Diagenode, #C15410195 (validated by ChIP in E(z)-KD in Zenk et al. 2017, Science and here by EED inhibition Ext. Data Fig. 11b)<br>H3K27ac Diagenode, #C15410196 (according to manufacturer validated for ChIP and NGS applications)<br>H3K36me3 Abcam AB9050 (according to manufacturer validated for ChIP and used in over 970 publications)<br>H3K4me1 Diagenode C15410194 (according to manufacturer validated for ChIP)<br>H3K4me3 Diagenode, #C15410003 (according to manufacturer used in more than 180 publications, validated for ChIP)<br>H3K9me3 Abcam ab176916 (validated by western blot and CUT&Tag in H3K9-methylation deficient embryos in Atinbayeva et al. 2024, EMBO J.)

Eukaryotic cell lines

Policy information about cell lines and Sex and Gender in Research

Cell line source(s)	WIBJ2 (WTSIi046-A, female) and HOIK (HPSI0314i-hoik_1, female) HipSci resource39 HCNP NeuroNA foundation,<br>CAU (female) HCNP NeuroNA foundation, Phenocell PC-1505<br>K562 (chronic myeloid leukemia in blast crisis), DSMZ ACC 10
Authentication	Cell lines used in the study were authenticated through comparing single nucleotide polymorphisms identified from single cell RNA and CUT&Tag to reference datasets.
Mycoplasma contamination	Regular PCR testing of all cell lines used in the study confirmed that they were free mycoplama contamination.
Commonly misidentified lines<br>(See ICLAC register)	None.

Animals and other research organisms

Policy information about studies involving animals; ARRIVE guidelines recommended for reporting animal research, and Sex and Gender in Research

Laboratory animals	Zebrafish embryos (wild-type Tupfel longfin/AB) were cultured 22 hours post fertilization. In general zebrafish were maintained at

Laboratory animals	the EPFL fish facility accredited by the the Service de la Consommation et des Affaires Vétérinaires of the canton of Vaud, Switzerland (authorization number VD-H23).
Wild animals	The study did not involve wild animals.
Reporting on sex	We did not identify the sex of the embryos
Field-collected samples	The study did not involve samples collected from the field.
Ethics oversight	Embryos were analysed before day 5 of embyonic development. They are covered by are covered under the general animal experiment license of the EPFL granted by the Service de la Consommation et de Affaires Vétérinaires of the canton of Vaud, Switzerland (authorization number VD-H23).

Note that full information on the approval of the study protocol must also be provided in the manuscript.

Plants

Seed stocks	not applicable
Novel plant genotypes	not applicable
Authentication	not applicable
