# Supplementary material for: A scalable Tn5-based method for genome-wide DNA methylation profiling in development and disease
Source: Nat Commun. 2026 May 22;17:6736. doi: 10.1038/s41467-026-73325-4 (PMC13385352; doi:10.1038/s41467-026-73325-4)
Supplement: Supplementary file 6 — Transparent Peer Review file [file 41467_2026_73325_MOESM6_ESM.pdf]

nature portfolio

Peer Review File

A scalable Tn5-based method for genome-wide DNA methylation profiling in development and disease

Corresponding Author: Professor Fides Zenk

This file contains all reviewer reports in order by version, followed by all author rebuttals in order by version.

Version 0:

Reviewer comments:

Reviewer #1

(Remarks to the Author)
The authors introduce MethCUT&Tag, a new whole-genome DNA cytosine methylation sequencing platform that employs a methylation-binding domain–fused Tn5 transposase to selectively tagment methylated DNA regions. They demonstrate that this approach is effective both for in-nucleus chromatin tagmentation and for purified DNA. When combined with bisulfite treatment, the method enables base-pair resolution profiling of DNA methylation with cost-efficient, minimal sequencing coverage. Finally, the authors apply MethCUT&Tag to study DNA methylation dynamics during brain organoid development, highlighting its potential for biological investigations.
Overall, the methylation-binding domain–fused Tn5 transposase represents an interesting new molecular tool for DNA methylation analysis with broad applications in epigenetics. In light of the rapid expansion of Tn5-based single-cell and spatial omics technologies (e.g., ATAC-seq, CUT&Tag), this tool could represent an important innovation by integrating DNA methylation as an additional modality for multi-omics approaches. However, I find that the current manuscript lacks several critical elements needed to facilitate the broad dissemination and adoption of MethCUT&Tag.

Major comments
1. One of the major concerns with CUT&Tag technology is the ATAC-seq-like background signal generated by Tn5 transposase. Although the authors examine background signals of untargeted Tn5 using purified DNA (Fig. 2), they do not assess this in a chromatin context. A direct comparison between 2XMBD2-Tn5 and untargeted Tn5 in an in-nucleus assay, with quantification of methyl-DNA-derived enrichment, is essential.
2. CUT&Tag has become widely adopted because of its efficiency with low cell numbers. The authors use relatively large inputs (100K–1M cells or 50 ng DNA). Demonstrating the robustness of MethCUT&Tag (not necessarily with bisulfite conversion) with lower inputs would significantly enhance its accessibility and impact.
3. In Fig. 1, the authors compare MethCUT&Tag with bisulfite-seq. However, they should also include a comparison with MeDIP, which is a more direct reference method, given that both rely on methyl-DNA-binding proteins.

Minor comments
4. A detailed protocol for MethCUT&Tag using isolated DNA is needed. This protocol is likely highly sensitive to the ratio of Tn5 transposase to input DNA.
5. Line 65: “The MBD Tn5 fusion proteins preferentially bound regions exhibiting high DNA methylation (>75%).” Please clarify what specific genomic regions these correspond to.
6. Fig. 2: “KO” is misleading, as the condition reflects DNMT1 inhibitor treatment rather than a genetic knockout.
7. Fig. 4d and Fig. S2h: Statistical details for the gene ontology analysis (e.g., P values) are missing and should be provided.
8. The authors should further discuss the potential of their technology in the context of emerging single-cell and spatial multi-omics approaches based on Tn5 transposase.

Reviewer #2

(Remarks to the Author)
This manuscript describes the development of a new method to detect DNA methylation, utilising CUT&Tag, which has so far been primarily applied to histone modifications and chromatin-binding proteins. The authors generated fusion proteins of Tn5 with various methyl-DNA-binding domains, all of which enable the detection of DNA methylation with higher efficiency than using an antibody directed towards 5mC. The specificity of the method was evaluated using a small molecule inhibitor

of the maintenance methyltransferase DNMT1. Finally, the method was applied to human brain organoids to assess changes in DNA methylation in a biologically relevant context.

Overall, the authors have presented evidence that the CUT&Tag-based detection of DNA methylation is effective and comparable to the detection of other epigenetic modifications by CUT&Tag. CUT&Tag is known for its high signal-to-noise ratio and efficiency on relatively small cell numbers, which the authors exploited here using DNA binding and Tn5 fusion proteins.

The authors propose that the new MethCUT&Tag approach may be applied to biomedical questions, offering scalability due to reduced costs resulting from lower sequence coverage compared to WGBS, which presents the current gold standard. One major limitation of the manuscript is that the authors have not sufficiently demonstrated the applicability of the method. Without a meaningful use case, this remains a theoretical option. Would the method be sensitive enough to detect biologically meaningful differences in a biomedical context?

Another major limitation is that the authors provided only a limited comparison to alternative methods for detecting DNA methylation. WGBS is only one of many ways. Other methods, such as RRBS, MethylationEPIC array and published methods (for example: doi.org/10.1038/s41592-025-02847-4; PMID 40999097) also provide specific advantages compared to WGBS. As the authors correctly state, resolution, cost, and throughput are relevant factors which have not been sufficiently addressed in comparison to these methods.

Given these limitations, without any novel biological insights, the manuscript appears a better fit for a method journal.

Additional points:

1) Information on biological and technical replicates should be provided for each experiment. Which iPSC line was used for which experiment? How comparable is the data between replicates? How many cells were used for iPSC and organoids?

2) Quantitative data should be presented on the different methyl-binding fusion proteins. How many regions overlapped or were unique to each fusion? What is known about these fusion proteins? Do they have any binding preferences?

3) The inhibitor experiment was used to assess the specificity of the method. The label “KO” is misleading. There are major differences between nuclei and genomic DNA. How can these be explained? To what extent is DNA methylation lost using the inhibitor conditions (based on WGBS)? Does the remaining signal indicate unspecific transposition or residual DNA methylation?

4) Additional data should be presented to justify the selection of 2×MBD2-Tn5. Moreover, it would be interesting to see the data for the 5mC antibody.

5) The DNA methylome of brain organoids has previously been described in Luo et al, Cell Rep 2026, and as such, the data presented in Figure 4 lack novelty. At the very least, the authors should benchmark the results obtained by MethCUT&Tag against this published data.

6) Images should be presented of the cells and organoids that went into the genomics experiments.

7) The raw data was not accessible to the reviewer for evaluation.

Reviewer #3

(Remarks to the Author)
This manuscript presents MethCUT&Tag, a promising new method that combines methylation-binding domain (MBD) fusion proteins with a hyperactive Tn5 transposase to target methylated regions of DNA for sequencing. The approach offers a potential powerful alternative for low-cost methylation profiling, and the data shown in human stem cells and organoid systems are convincing. The correlation with bisulfite sequencing is encouraging, and the demonstration of methylation dynamics during brain organoid development highlights the method’s biological relevance. Overall, I am positive about the work and its potential impact, but there are several points that should be clarified or expanded to strengthen the manuscript.

The authors should briefly discuss how MethCUT&Tag fits within the landscape of current methylation-profiling technologies. In particular, single-molecule sequencing platforms such as PacBio and the more accessible Oxford Nanopore (ONT) now allow direct methylation detection and show strong correlation with WGBS. Mentioning these would provide a more balanced overview of existing approaches.

Since the method also relies on MBD binding, previous enrichment approaches such as MethylCap-seq (e.g., Brinkman et al.) should be cited. It would be helpful to clarify how MethCUT&Tag improves on these earlier method, whether in terms of resolution, input requirements, workflow simplicity, or the ability to work directly on chromatin.

The authors should provide an estimate of how many peaks are typically identified per sample and what fraction of the genome they cover. A simple genomic annotation breakdown, showing the proportions mapping to promoters, gene bodies, CpG islands, and enhancers, would make the data easier to interpret.

Are there peaks that lack CpG dinucleotides altogether? If so, they may reflect sequence preferences or biases of the MBD domains rather than genuine methylation signals. Including a short analysis or control addressing this would strengthen confidence in specificity.

The manuscript should clearly state the recommended DNA or cell input amounts. If MethCUT&Tag performs well with very low input, this would be a notable advantage compared with WGBS or EM-seq and should be highlighted.

Some discussion of the method's potential limitations would make the paper more balanced. WGBS, EM-seq, and long-read technologies are largely organism-agnostic, whereas MethCUT&Tag uses human MBD domains. It would be useful to clarify whether the technique has been tested or is expected to be compatible with mouse DNA, and whether it could be adapted for other species such as zebrafish, Xenopus and other non mammalian vertebrates. This would help readers understand how broadly the approach can be applied.

Version 1:

Reviewer comments:

Reviewer #1

(Remarks to the Author)
The authors have satisfactorily addressed my previous concerns regarding specificity and benchmarking, and I appreciate the additional experiments and clarifications provided in the revised manuscript.

The inclusion of a single-cell implementation of CmeCUT&Tag is interesting and represents a potentially important extension of the method. However, upon closer examination of the single-cell data, I have several concerns regarding the strength of the conclusions drawn from this single-cell analysis.

First, the current experiment relies on a mixture of two highly distinct cell types (K562 leukemia cells and iPSCs), which are epigenetically very different. While the observed separation between these populations demonstrates compatibility with droplet-based workflows, it does not provide evidence that the method can resolve more subtle differences, such as closely related cell states or heterogeneous populations within tissues. As such, I would encourage the authors to temper their claims and restrict their conclusions to demonstrating compatibility with single-cell workflows.

Second, the clustering results themselves appear relatively coarse. In Fig. 2g, both clusters contain a substantial fraction of the opposing cell type (i.e., K562 cells in cluster 0 and iPSCs in cluster 1), suggesting that separation is not clean but rather reflects a general trend. This raises concerns about signal sparsity, noise, or insufficient per-cell information content, and should be discussed more explicitly.

Third, the overall recovery appears low. The experiment is described as starting from approximately 2 million cells, yet only ~1,100 cells are ultimately recovered, and ~500 cells per population are retained after filtering. It is unclear how many nuclei were loaded into the 10x Chromium controller and what the expected versus observed recovery rates were. Providing a detailed accounting of cell numbers at each step (input, loading, recovery, filtering) would greatly improve transparency and allow proper assessment of method efficiency.

Finally, key quality control metrics typically expected for single-cell chromatin assays are not clearly reported. For example, it is unclear whether FRiP scores, duplication rates, or other per-cell quality metrics were assessed and used for filtering. In addition, the number of biological or technical replicates for the single-cell experiment should be explicitly stated.

Overall, while the single-cell extension is interesting, the current data should be presented more cautiously as a proof-of-concept demonstration of compatibility with droplet-based workflows, rather than as evidence of a mature or robust single-cell DNA methylation profiling platform. Clarification of the above points and a more balanced discussion would substantially strengthen this part of the manuscript.

(Remarks on code availability)
I did not run the code myself, but it appears to be well-structured and self-explanatory.

Reviewer #2

(Remarks to the Author)
In the revised manuscript, the authors provide several additional new data and analyses that significantly strengthen the work. Specifically, the authors show the applicability of the method by using it to classify different brain tumor types and by demonstrating its applicability to study zebrafish development. Moreover, the authors now provide more detailed benchmarking of the new CmeCUT&Tag method against existing approaches. The titration of input material further increases the applicability of the new method. The raw data is now also accessible via the GEO repository. In summary, my concerns have been satisfactorily addressed, and I recommend the manuscript for publication.

(Remarks on code availability)

Reviewer #3

(Remarks to the Author)
The authors have gone above and beyond in addressing the reviewers' comments. I congratulate them on their excellent work and fully support the publication of this manuscript.

(Remarks on code availability)

Open Access This Peer Review File is licensed under a Creative Commons Attribution 4.0 International License, which permits use, sharing, adaptation, distribution and reproduction in any medium or format, as long as you give appropriate credit to the original author(s) and the source, provide a link to the Creative Commons license, and indicate if changes were made.
In cases where reviewers are anonymous, credit should be given to 'Anonymous Referee' and the source.
The images or other third party material in this Peer Review File are included in the article's Creative Commons license, unless indicated otherwise in a credit line to the material. If material is not included in the article's Creative Commons license and your intended use is not permitted by statutory regulation or exceeds the permitted use, you will need to obtain permission directly from the copyright holder.
To view a copy of this license, visit https://creativecommons.org/licenses/by/4.0/

We sincerely thank the reviewers for their thoughtful, constructive, and insightful evaluation of our manuscript. We appreciate the positive assessment of the conceptual advance and the detailed suggestions aimed at strengthening the methodological rigor, biological applicability, and positioning of our approach within the broader epigenomics landscape. In response, we have performed several new experiments, expanded comparative benchmarking against alternative methylation-profiling technologies, clarified methodological aspects, and substantially revised the manuscript for clarity and balance. For clarity and consistency, we have also renamed the method C^{me}CUT&Tag from MethCUT&Tag throughout the revised manuscript. We believe these additions and revisions significantly strengthen the study.

REVIEWER COMMENTS

Reviewer #1 (Remarks to the Author):

The authors introduce MethCUT&Tag, a new whole-genome DNA cytosine methylation sequencing platform that employs a methylation-binding domain–fused Tn5 transposase to selectively tagment methylated DNA regions. They demonstrate that this approach is effective both for in-nucleus chromatin tagmentation and for purified DNA. When combined with bisulfite treatment, the method enables base-pair resolution profiling of DNA methylation with cost-efficient, minimal sequencing coverage. Finally, the authors apply MethCUT&Tag to study DNA methylation dynamics during brain organoid development, highlighting its potential for biological investigations. Overall, the methylation-binding domain–fused Tn5 transposase represents an interesting new molecular tool for DNA methylation analysis with broad applications in epigenetics. In light of the rapid expansion of Tn5-based single-cell and spatial omics technologies (e.g., ATAC-seq, CUT&Tag), this tool could represent an important innovation by integrating DNA methylation as an additional modality for multi-omics approaches. However, I find that the current manuscript lacks several critical elements needed to facilitate the broad dissemination and adoption of MethCUT&Tag.

Thank you for the thoughtful and encouraging evaluation of the manuscript and for recognizing C^{me}CUT&Tag as a promising tool with broad applicability and strong potential for integration into emerging multi-omic platforms. The constructive feedback provided has been highly valuable and has guided improvements to the clarity, completeness, and overall accessibility of the manuscript.

Major comments

1. One of the major concerns with CUT&Tag technology is the ATAC-seq-like background signal generated by Tn5 transposase. Although the authors examine background signals of untargeted Tn5 using purified DNA (Fig. 2), they do not assess this in a chromatin context. A direct comparison between 2XMBD2-Tn5 and untargeted Tn5 in an in-nucleus assay, with quantification of methyl-DNA-derived enrichment, is essential.

Thank you for raising this important point regarding potential background binding of Tn5 to open chromatin. We fully agree that this is a critical control. In the C^{me}CUT&Tag protocol, reactions are performed at high salt concentrations (300 mM NaCl) to counteract Tn5's intrinsic tendency to bind accessible chromatin regions. Under these conditions, Tn5 alone generates libraries with extremely low yield, consistent with minimal nonspecific binding. We have now sequenced these control libraries and incorporated the results into Fig. 2a, which demonstrates that untargeted Tn5 does not recapitulate the peak pattern of our MBD2 coupled construct even *in situ* in nuclear chromatin.

To further address your concern, we have incorporated published ATAC-seq profiles into the ChromHMM characterization (Fig. 1d) and now include IGV tracks comparison between ATAC-seq and C^{me}CUT&Tag in Extended Data Fig. 2b. These analyses demonstrate that ATAC-seq patterns are clearly distinct from C^{me}CUT&Tag signals and from genomic regions marked by high levels of DNA methylation.

As an additional specificity control, we coupled Tn5 to the CXXC domain of MLL1, which produces profiles that are markedly different from those obtained with MBD2-coupled Tn5. These new data have now been added to Fig. 1d and Extended Data Fig. 2f-i.

In addition, inhibitor experiments performed on isolated nuclei provide further support for the specificity of MBD–Tn5 binding. Upon inhibitor treatment, MBD–Tn5 peaks are globally and markedly reduced, indicating that the detected signal does not arise from intrinsic Tn5 activity. If substantial nonspecific Tn5 binding were present, signal would be expected to persist following the inhibitor treatment, which is not observed.

2. CUT&Tag has become widely adopted because of its efficiency with low cell numbers. The authors use relatively large inputs (100K–1M cells or 50 ng DNA). Demonstrating the robustness of MethCUT&Tag (not necessarily with bisulfite conversion) with lower inputs would significantly enhance its accessibility and impact.

Thank you for highlighting the importance of low-input performance. To directly address this point, we performed a serial dilution experiment to assess the input requirements of C^{me}CUT&Tag. We demonstrate that robust profiles can be obtained with inputs as low as 5 ng of DNA or 50k cells without any low-input–specific adaptations to the protocol. These new data are now included in Extended Data Fig. 3b and 3g and described in the Methods. In addition, a comprehensive supplementary table has been added summarizing metadata for all analysed samples, including input amounts and resulting library concentration.

3. In Fig. 1, the authors compare MethCUT&Tag with bisulfite-seq. However, they should also include a comparison with MeDIP, which is a more direct reference method, given that both rely on methyl-DNA-binding proteins.

Thank you for the suggestion to include a comparison with MeDIP-based approaches. We have now included direct comparison with published datasets in Fig. 1d, showing that C^{me}CUT&Tag signals closely match those obtained by MBD-seq and 5mC-MeDIP in human embryonic stem cells and neurospheres.

In addition, we show in Extended Data Fig. 4 that genome-wide signal distributions are highly similar across methods, both globally and at representative loci, as illustrated by IGV browser snapshots. Notably, C^{me}CUT&Tag and MBD-Seq exhibit slightly stronger enrichment toward highly methylated regions compared to MeDIP, consistent with the known binding properties of MBD-based approaches.

We have further benchmarked species transferability of the human MBD2-Tn5 to zebrafish chromatin by comparing C^{me}CUT&Tag to MethylCap-Seq in a new experiment, again observing highly concordant enrichment profiles (Extended Data Fig. 8).

Finally, a comparative table summarizing key features of these technologies, including input requirements, library preparation strategies, and cost with C^{me}CUT&Tag has been added to this manuscript as Supplementary Table 3.

Minor comments
4. A detailed protocol for MethCUT&Tag using isolated DNA is needed. This protocol is likely highly sensitive to the ratio of Tn5 transposase to input DNA.

Thank you very much for pointing this out. We have revised the Methods section and added a dedicated subsection describing the C^{me}CUT&Tag protocol for isolated DNA and have added a graphical outline in Extended Data 1c. In addition, we performed titration experiments to provide practical guidance on reagent usage across different input types. These new data are presented in Extended Data Fig. 3a and 3f.

We found that 600–1500 ng of MBD2-Tn5 reliably fragments nuclei derived from 200,000 cells, whereas 300–600 ng of MBD2-Tn5 is optimal for 25 ng of isolated DNA. Notably, using 1500 ng of MBD2-Tn5 with 25 ng of isolated DNA resulted in over-fragmentation and reduced library preparation efficiency, highlighting the importance of dosage optimization based on the input type.

5. Line 65: “The MBD Tn5 fusion proteins preferentially bound regions exhibiting high DNA methylation (>75%).” Please clarify what specific genomic regions these correspond to.

Thank you for pointing this out. We have now performed additional analyses to address this point and added new data in Extended Data Fig. 2e. In this figure, we annotate the binding profiles of all MBD fusion constructs using ChIPseeker to provide a systematic genomic feature classification.

In addition, we applied the ChromHMM-based analysis to define regions with high DNA methylation (>75% methylation) based on integrated WGBS and RRBS datasets. We have updated the figure legend to clarify this definition. These highly methylated regions are predominantly associated with heterochromatin, transcribed gene bodies, enhancer elements, and Polycomb-repressed domains.

6. Fig. 2: “KO” is misleading, as the condition reflects DNMT1 inhibitor treatment rather than a genetic knockout.

We apologise for the oversight and thank you for seeing this. We have corrected it in the revised figures.

7. Fig. 4d and Fig. S2h: Statistical details for the gene ontology analysis (e.g., P values) are missing and should be provided.

Thank you for pointing this out. We have corrected this in the revised figures and added Supplementary Table 2 with the complete analysis output.

8. The authors should further discuss the potential of their technology in the context of emerging single-cell and spatial multi-omics approaches based on Tn5 transposase.

Thank you for this insightful comment. This comment motivated us to directly test the compatibility of our MBD2–Tn5 fusion protein with 10x Genomics single-cell workflows. We performed scC^{me}CUT&Tag on a mixed population of human iPS cells with K562 leukemia cells. As shown in the new Fig. 2e-g, scC^{me}CUT&Tag yields high-quality data that can robustly resolve the major cell types within the mixture.

In Addition, we have also expanded the Discussion (line 233-238) to address the broader potential of C^{me}CUT&Tag for integration into emerging single-cell and future spatial multi-omics platforms that leverage Tn5-based chemistries.

Reviewer #2 (Remarks to the Author):

This manuscript describes the development of a new method to detect DNA methylation, utilising CUT&Tag, which has so far been primarily applied to histone modifications and chromatin-binding proteins. The authors generated fusion proteins of Tn5 with various methyl-DNA-binding domains, all of which enable the detection of DNA methylation with higher efficiency than using an antibody directed towards 5mC. The specificity of the method was evaluated using a small molecule inhibitor of the maintenance methyltransferase DNMT1. Finally, the method was applied to human brain organoids to assess changes in DNA methylation in a biologically relevant context.

Overall, the authors have presented evidence that the CUT&Tag-based detection of DNA methylation is effective and comparable to the detection of other epigenetic modifications by CUT&Tag. CUT&Tag is known for its high signal-to-noise ratio and efficiency on relatively small cell numbers, which the authors exploited here using DNA binding and Tn5 fusion proteins.

The authors propose that the new MethCUT&Tag approach may be applied to biomedical questions, offering scalability due to reduced costs resulting from lower sequence coverage compared to WGBS, which presents the current gold standard. One major limitation of the manuscript is that the authors have not sufficiently demonstrated the applicability of the method. Without a meaningful use case, this remains a theoretical option. Would the method be sensitive enough to detect biologically meaningful differences in a biomedical context?

Another major limitation is that the authors provided only a limited comparison to alternative methods for detecting DNA methylation. WGBS is only one of many ways. Other methods, such as RRBS, MethylationEPIC array and published methods (for example: doi.org/10.1038/s41592-025-02847-4; PMID 40999097) also provide specific advantages compared to WGBS. As the authors correctly state, resolution, cost, and throughput are relevant factors which have not been sufficiently addressed in comparison to these methods.

Given these limitations, without any novel biological insights, the manuscript appears a better fit for a method journal.

Thank you very much for your thoughtful and constructive review. We appreciate your clear summary of the manuscript and your recognition of the novelty and potential of our CUT&Tag-based approach for detecting DNA methylation. We fully agree that demonstrating practical biological applicability and benchmarking against alternative methods are essential for evaluating the utility and the impact of the approach.

To address these important points, we have performed several new experiments and significantly expanded the revised manuscript:

Classification of tumor subtypes: We have now performed additional experiments demonstrating that C^{me}CUT&Tag–EM can be used to subclassify brain tumor samples, enabling accurate assignment to established methylation-based tumor classes (Fig. 5). These results illustrate that C^{me}CUT&Tag is sufficiently sensitive to resolve biologically and clinically meaningful methylation differences and highlight its relevance for biomedical and diagnostic applications.

Applicability across biological systems: We now include data demonstrating successful application of C^{me}CUT&Tag in *Danio rerio* (zebrafish embryos), highlighting the method's transferability across species and its ability to capture complex methylation landscapes in a biologically relevant vertebrate model (see Extended Data Fig. 8).

Benchmarking against alternative technologies: We now provide a more detailed benchmarking of C^{me}CUT&Tag against not only WGBS, RRBS and MethylationEPIC array (as in Fig. 1d, Fig. 3e, and Extended Fig. 4) but also MethylCap, MBD-Seq and MeDIP, including a comparison of resolution, cost, input requirements, and scalability (see updated Discussion line 228-236 and Extended Data Fig. 4 and 8 as well as Supplementary Table 3).

In situ profiling and scalability: We would also like to highlight that, unlike traditional enrichment-based methods, C^{me}CUT&Tag can be performed directly in nuclei and is compatible with droplet-based single-cell genomics workflows. In the revised manuscript, we now demonstrate robust compatibility with a droplet microfluidics platform (Fig. 2e-g), enabling single-cell DNA methylation profiling in mixed human cell populations.

Together, these additions demonstrate that C^{me}CUT&Tag is not only conceptually innovative but also technically robust, scalable, broadly applicable, and biologically informative.

We agree that establishing biological relevance is essential. Previous studies using MBD-based enrichment approaches including MethylCap-seq and 5mC-MeDIP demonstrated that methylation profiling can reveal biologically meaningful changes across development, disease, and cancer, providing a strong foundation for enrichment-based strategies. Building on this framework, C^{me}CUT&Tag preserves methylation-specific targeting while significantly improving cost-efficiency, workflow speed, input requirements, and scalability. By directly fusing MBD domains to Tn5, our approach eliminates end repair and adapter ligation steps, enabling rapid library preparation and straightforward integration with single-cell platforms. We therefore view C^{me}CUT&Tag as both a technical advance and a practical platform for biological and translational epigenomics.

Additional points:

1) Information on biological and technical replicates should be provided for each experiment. Which iPSC line was used for which experiment? How comparable is the data between replicates? How many cells were used for iPSC and organoids?

Thank you for pointing this out. We have now added a comprehensive metadata table detailing the iPSC lines used, input amounts, and replicate numbers (Supplementary Table 1). In addition, we have updated all figure legends to specify the number of biological replicates and to explicitly reference the Pearson correlation analysis shown in Extended Data Fig. 2a and line 72-73.

2) Quantitative data should be presented on the different methyl-binding fusion proteins. How many regions overlapped or were unique to each fusion? What is known about these fusion proteins? Do they have any binding preferences?

Thank you for bringing this up. We agree that this was not adequately highlighted in the previous version of the manuscript. We have therefore included a detailed description of the analysis and the choice of domains in the revised text (line 58-69 and 75-83). We have performed additional analysis on the peaks (Extended Data Fig. 2d-e) and also refined the figure legend of Extended Data Fig. 2d.

3) The inhibitor experiment was used to assess the specificity of the method. The label “KO” is misleading. There are major differences between nuclei and genomic DNA. How can these be explained? To what extent is DNA methylation lost using the inhibitor conditions (based on WGBS)? Does the remaining signal indicate unspecific transposition or residual DNA methylation?

Thank you for these important observations. We apologize for the misleading “KO” label in the original figure and have corrected it to reflect the appropriate experimental condition, namely pharmacological inhibition of DNMT1 using GSK3484862.

Previous characterization of this inhibitor demonstrated a substantial global reduction in DNA methylation, while genome-wide CpG methylation decreasing from approximately ~70% to ~17% (PMID: 34906184) comparable to the genetic depletion of DNMT3a/b or DNMT1. To directly quantify methylation levels in our experimental system, we performed EM-Seq, a method to enzymatically convert unmethylated Cytosin to Uracil, on the samples used for the C^{me}CUT&Tag and observed a reduction in global DNA methylation from 80% to 20%. These data are now included in Extended Data Fig. 5a. Consistent with this, immunofluorescence staining, confirms the presence of residual DNA-methylation following inhibitor treatment (Extended Data Fig. 5b).

To address your concern and determine whether the remaining C^{me}CUT&Tag signal reflects nonspecific transposition or residual methylation, we re-analyzed our data with a focus on CpG content. We found that regions retaining C^{me}CUT&Tag signal are significantly enriched for CpG dinucleotides (Extended Data Fig. 5c-d), supporting the interpretation that MBD2-Tn5 continues to bind specifically to loci with residual DNA methylation rather than to random genomic regions.

With respect to the observed differences between nuclei and genomic DNA (gDNA), profiles were generated over the same peak set to enable direct comparison. We consistently observe higher signal intensity and peak numbers in gDNA relative to nuclei (Extended Data Fig. 3h), which likely reflects increased accessibility of methylated sites in deproteinized DNA. In chromatin, nucleosome occupancy and steric constraints may limit access to a subset of methylated loci. Although both gDNA and nuclei samples show reduced signal upon inhibitor treatment, the residual signal is slightly higher in gDNA, consistent with enhanced accessibility of the remaining methylated regions and the high affinity of MBD2-Tn5 for CpG-dense regions. We are now explaning this in the text (line 126-132).

4) Additional data should be presented to justify the selection of 2×MBD2-Tn5. Moreover, it would be interesting to see the data for the 5mC antibody.

Thank you for pointing this out. We have now expanded this discussion in the main text (line 58–69) and provided a more detailed comparative analysis in line 75–83 and 111–116. Additionally, we now include an IGV track of the 5mC CUT&Tag data in Extended Data Fig. 2b and more details in Supplementary Table 1. These data highlight that libraries generated using 5mC antibody in *in situ* nuclei yield very low signal with no discernible genomic enrichment. Consistently, peak calling with MACS3 fails to identify significant peaks (Extended Data Fig. 2d).

This behavior likely reflects a technical limitation of antibody-based 5mC detection: conventional 5mC immunoprecipitation approaches typically require denatured, single-stranded DNA, which is not achievable in intact nuclei under *in situ* conditions. Conversely, antibody-based CUT&Tag is not readily compatible with isolated DNA, as key protocol steps such as washing are not feasible without immobilizing the DNA. Together, these constraints highlight the need for alternative strategies, such as our MBD–Tn5 fusion strategy, that enable efficient methylation profiling both *in situ* and on purified DNA.

5) The DNA methylome of brain organoids has previously been described in Luo et al, Cell Rep 2026, and as such, the data presented in Figure 4 lack novelty. At the very least, the authors should benchmark the results obtained by MethCUT&Tag against this published data.

Thank you for pointing this out. We have benchmarked C^{me}CUT&Tag extensively against Luo et al, Cell Rep 2016, and observe high concordance between WGBS sequencing and C^{me}CUT&Tag-enriched regions in iPSC/human ES cells (Fig. 1c-f, Extended Data Fig. 2b, f, h, j, Extended Data Fig. 3k, Extended Data Fig. 4a-c, Extended Data Fig. 6a).

We have now expanded this comparison to include their organoid datasets (Extended Data Fig. 7b-c). Despite differences in developmental stages, we observe a consistent trend, in dynamics of DNA methylation patterns including both gains and losses across organoid differentiation.

In addition, we now highlight representative loci showing dynamic methylation changes based on a recently available dataset (bioRxiv: 2025.10.01.679721), where the complete data are not yet available for formal genome-wide analysis.

6) Images should be presented of the cells and organoids that went into the genomics experiments.

Thank you for your suggestion. We have now included them in Extended Data Fig. 1c and Extended Data Fig. 7a, respectively.

7) The raw data was not accessible to the reviewer for evaluation.

We apologize for this and have now prepared everything on GEO with a reviewer link.

Here is the link and the reviewer token to our GEO repository:

https://www.ncbi.nlm.nih.gov/geo/query/acc.cgi?acc=GSE320203

Token: wvwjwwsshzurjez

Reviewer #3 (Remarks to the Author):

This manuscript presents MethCUT&Tag, a promising new method that combines methylation-binding domain (MBD) fusion proteins with a hyperactive Tn5 transposase to target methylated regions of DNA for sequencing. The approach offers a potential powerful alternative for low-cost methylation profiling, and the data shown in human stem cells and organoid systems are convincing. The correlation with bisulfite sequencing is encouraging, and the demonstration of methylation dynamics during brain organoid development highlights the method's biological relevance. Overall, I am positive about the work and its potential impact, but there are several points that should be clarified or expanded to strengthen the manuscript.

The authors should briefly discuss how MethCUT&Tag fits within the landscape of current methylation-profiling technologies. In particular, single-molecule sequencing platforms such as PacBio and the more accessible Oxford Nanopore (ONT) now allow direct methylation detection and show strong correlation with WGBS. Mentioning these would provide a more balanced overview of existing approaches.

Thank you very much for your positive assessment of our work and for highlighting its potential impact. We appreciate your thoughtful suggestion to better contextualize C^{me}CUT&Tag within the broader landscape of DNA methylation profiling technologies.

We have now expanded the Discussion (line 220–223) to provide a more balanced overview of current approaches, including single-molecule long-read sequencing platforms such as PacBio and Oxford Nanopore. In addition, we have generated a comprehensive comparative table positioning C^{me}CUT&Tag alongside other methylation-profiling technologies with respect to resolution, input requirements, cost, scalability, and technical complexity (Supplementary Table 3)

Since the method also relies on MBD binding, previous enrichment approaches such as MethylCap-seq (e.g., Brinkman et al.) should be cited. It would be helpful to clarify how MethCUT&Tag improves on these earlier method, whether in terms of resolution, input requirements, workflow simplicity, or the ability to work directly on chromatin.

Thanks a lot for your suggestion. We have now included comparisons to MeDIP, MethylCap-Seq, and MiGS in the comparative table (Supplementary Table 3) and expanded the discussion accordingly (line 58-59, 228-236). We highlight that the main advantages of C^{me}CUT&Tag include reduced cost per sample, low input requirement (now shown in Extended Data Fig. 3a-b and f-g), simplification of the workflow, elimination of end repair and adapter ligation steps, and the ability to operate directly with nuclei in situ. This *in situ* compatibility further enables adaptation to droplet-based single cell technologies, as demonstrated in Fig. 2e-g.

The authors should provide an estimate of how many peaks are typically identified per sample and what fraction of the genome they cover. A simple genomic annotation breakdown, showing the proportions mapping to promoters, gene bodies, CpG islands, and enhancers, would make the data easier to interpret.

Thanks a lot for your comment. We agree that this was not highlighted well enough in the previous version and we have now dedicated more space to this in the text (line 75-84 and 123-136) and figures (Extended Data Fig. 2d and 3h). We have also performed additional analysis to annotate the genomic regions in addition to the ChromHMM annotation (Extended Data Fig. 2e and 4a).

Are there peaks that lack CpG dinucleotides altogether? If so, they may reflect sequence preferences or biases of the MBD domains rather than genuine methylation signals. Including a short analysis or control addressing this would strengthen confidence in specificity.

Thank you for this suggestion. To assess potential nonspecific binding by MBD2-Tn5, we analyzed the residual signal observed following DNA methylation inhibitor treatment. If this signal were driven by nonspecific transposition, it would not be expected to preferentially localize to CpG dense regions. Instead, the remaining signal is strongly enriched for CpG-rich loci, arguing against non-specific Tn5 activity (Extended Data Fig. 5c-d). We generally observe a high CG content in C^{me}CUT&Tag peaks (Extended Data Fig. 3j and 8d).

Consistent with this, global DNA methylation levels only decrease from ~70% to ~17% following inhibitor treatment, as reported previously (PMID: 34906184) and confirmed by our EM-Seq measurements (~80% to ~20%) (Extended Data Fig. 5a-b). We therefore interpret the residual C^{me}CUT&Tag signal as originating primarily from genomic regions that retain DNA methylation despite pharmacological inhibition. This interpretation is further supported by the absence of signal in CpG islands classified as unmethylated (Extended Data Fig. 2g).

The manuscript should clearly state the recommended DNA or cell input amounts. If MethCUT&Tag performs well with very low input, this would be a notable advantage compared with WGBS or EM-seq and should be highlighted.

Thanks again for highlighting this point. We have now performed additional titration experiments to determine the optimal input amount requirements for C^{me}CUT&Tag (Extended Data Fig. 3a-b and f-g) and revised the corresponding sections of the manuscript (line 114-117 and 121-122). In addition, we have included Supplementary Table 3 to provide a comparative overview of input requirements across methylation-profiling technologies as well as all experiments analyzed in the manuscript (Supplementary Table 1).

Some discussion of the method's potential limitations would make the paper more balanced. WGBS, EM-seq, and long-read technologies are largely organism-agnostic, whereas MethCUT&Tag uses human MBD domains. It would be useful to clarify whether the technique has been tested or is expected to be compatible with mouse DNA, and whether it could be adapted for other species such as zebrafish, Xenopus and other non mammalian vertebrates. This would help readers understand how broadly the approach can be applied.

Thanks for your suggestion. We have now balanced the Discussion accordingly (line 241-244). In addition, this comment prompted us to directly test cross-species transferability, and we successfully generated DNA methylation profiles in zebrafish that closely match published MethylCap-Seq Data and whole-genome methylation datasets (Extended Data Fig. 8). Together, these results suggest that C^{me}CUT&Tag is transferable across species.

Reviewer #2 (Remarks to the Author):

This manuscript describes the development of a new method to detect DNA methylation, utilising CUT&Tag, which has so far been primarily applied to histone modifications and chromatin-binding proteins. The authors generated fusion proteins of Tn5 with various methyl-DNA-binding domains, all of which enable the detection of DNA methylation with higher efficiency than using an antibody directed towards 5mC. The specificity of the method was evaluated using a small molecule inhibitor of the maintenance methyltransferase DNMT1. Finally, the method was applied to human brain organoids to assess changes in DNA methylation in a biologically relevant context.

Overall, the authors have presented evidence that the CUT&Tag-based detection of DNA methylation is effective and comparable to the detection of other epigenetic modifications by CUT&Tag. CUT&Tag is known for its high signal-to-noise ratio and efficiency on relatively small cell numbers, which the authors exploited here using DNA binding and Tn5 fusion proteins.

The authors propose that the new MethCUT&Tag approach may be applied to biomedical questions, offering scalability due to reduced costs resulting from lower sequence coverage compared to WGBS, which presents the current gold standard. One major limitation of the manuscript is that the authors have not sufficiently demonstrated the applicability of the method. Without a meaningful use case, this remains a theoretical option. Would the method be sensitive enough to detect biologically meaningful differences in a biomedical context?

Another major limitation is that the authors provided only a limited comparison to alternative methods for detecting DNA methylation. WGBS is only one of many ways. Other methods, such as RRBS, MethylationEPIC array and published methods (for example: doi.org/10.1038/s41592-025-02847-4; PMID 40999097) also provide specific advantages compared to WGBS. As the authors correctly state, resolution, cost, and throughput are relevant factors which have not been sufficiently addressed in comparison to these methods.

Given these limitations, without any novel biological insights, the manuscript appears a better fit for a method journal.

Thank you very much for your thoughtful and constructive review. We appreciate your summary of the manuscript and your recognition of the novelty and potential of our CUT&Tag-based approach for detecting DNA methylation. We fully agree that demonstrating practical applicability and benchmarking against alternative methods are essential for assessing the method's utility and impact.

To address these important points, we have performed several new experiments and significantly expanded the revised manuscript:

Applicability in diverse biological systems: We now include data demonstrating successful application of C^{me}CUT&Tag in *Danio rerio* (zebrafish embryos), highlighting the method's transferability across species and its ability to detect complex methylation patterns in a biologically relevant vertebrate model (see Extended Data Fig. 8).

Comparison to alternative technologies: We now provide a more detailed benchmarking of C^{me}CUT&Tag against not only WGBS, RRBS and MethylationEPIC array (as in Fig. 1d and Extended Fig. 4a) but also MethylCap, MBD-Seq and MeDIP, including a comparison of resolution, cost, input requirements, and scalability (see updated Discussion and Extended Data Fig. 4 and Supplementary Table 3).

In situ profiling and scalability: We would also like to highlight that, unlike traditional enrichment-based methods, C^{me}CUT&Tag can be performed directly in nuclei and is compatible with droplet-based single-cell genomics workflows. In the revised manuscript, we now demonstrate successful adaptation of the method to a droplet microfluidics platform, enabling single-cell DNA methylation profiling in mixed human cell populations.

Together, these additions help to demonstrate that C^{me}CUT&Tag is not only conceptually innovative but also technically robust, scalable, and biologically informative.

We agree that demonstrating biological relevance is essential. It is important to note that the biological meaningfulness of mapping DNA methylation using methyl-binding domain (MBD)-based methods such as MethylCap-seq but also 5mC-MeDIP has already been well established in the literature, where they have been successfully used to identify methylation changes across development, disease, and cancer subtypes. Building

on this, our C^{me}CUT&Tag approach preserves the core specificity of MBD-based enrichment while significantly improving cost-efficiency, workflow speed, input requirements, and scalability. Specifically, by fusing MBD domains directly to Tn5, we eliminate the need for end repair and adapter ligation, enabling fast and scalable library preparation. Furthermore, our method is now compatible with droplet-based single-cell workflows, opening new possibilities for high-resolution, high-throughput epigenetic profiling. We believe this combination of established biological interpretability and technical advancement makes C^{me}CUT&Tag particularly powerful for both fundamental and translational applications.

Additional points:

1) Information on biological and technical replicates should be provided for each experiment. Which iPSC line was used for which experiment? How comparable is the data between replicates? How many cells were used for iPSC and organoids?

Thank you for pointing this out. We have now generated a comprehensive table of metadata to add information on iPSC lines, input amounts, and replicate numbers (Supplementary Table 1). We have further added the number of replicates to all legends and refer now even more clearly to the Figure showing the Pearson's correlation of the replicates in Extended Data Fig. 2a.

2) Quantitative data should be presented on the different methyl-binding fusion proteins. How many regions overlapped or were unique to each fusion? What is known about these fusion proteins? Do they have any binding preferences?

Thank you for bringing this up. We agree that this was not adequately highlighted in the previous version of the manuscript. We have therefore included a detailed description of the analysis and the choice of domains in the revised text (Line 55-65 and 73-80). We have performed additional analysis on the peaks (Extended Data Fig. 2d-e) and also refined the figure legend of Extended Data Fig. 2d.

3) The inhibitor experiment was used to assess the specificity of the method. The label "KO" is misleading. There are major differences between nuclei and genomic DNA. How can these be explained? To what extent is DNA methylation lost using the inhibitor conditions (based on WGBS)? Does the remaining signal indicate unspecific transposition or residual DNA methylation?

Thank you for these important observations. We apologize for the misleading label "KO" in the figure and have now corrected it to reflect the appropriate experimental condition, which involves pharmacological inhibition of DNMT1 using GSK3484862.

In the original publication characterizing this inhibitor (PMID: 34906184), treatment led to a substantial global reduction in DNA methylation, decreasing genome-wide CpG methylation levels from ~70% to ~17%. This degree of depletion is comparable to genetic knockouts of DNMT3a/b or DNMT1 and effectively models global DNA hypomethylation. We have now performed EM-Seq on the samples used for the C^{me}CUT&Tag and can recapitulate the drop in DNA methylation from 80% to 20%. This data is now included in Extended Data Fig. 5a. We have also performed immunofluorescence staining, which confirm residual DNA-methylation upon inhibitor treatment (Extended Data Fig. 5b).

To address your concern regarding the residual signal after inhibitor treatment, we re-analyzed our data with a focus on CpG content. We found that regions retaining C^{me}CUT&Tag signal are significantly enriched for CpG dinucleotides (Extended Data Fig. 5c-d). The observed CpG enrichment supports the interpretation that MBD2-Tn5 continues to bind specifically to loci with residual DNA methylation rather than to random genomic regions.

Regarding the observed differences between nuclei and genomic DNA (gDNA):

The plots for nuclei and gDNA are generated over the same set of peaks for comparability. We consistently observe a higher signal intensity and peak numbers in gDNA compared to nuclei (Extended Data Fig. 3h), likely reflecting increased physical accessibility of methylated sites in deproteinized DNA. In chromatin, steric hindrance or nucleosome positioning may limit the fusion protein's access to some methylated loci. While both gDNA and nuclei samples show reduced signal upon inhibitor treatment, the residual signal in gDNA is slightly higher, which we attribute to enhanced accessibility of the remaining methylated sites and the high affinity of MBD2-Tn5 for CpG-dense regions.

4) Additional data should be presented to justify the selection of 2×MBD2-Tn5. Moreover, it would be interesting to see the data for the 5mC antibody.

We have now expanded this point in the main text (lines 55–65) and provided a more in-depth comparative analysis in lines 72–80 and 110–115. Additionally, we have included an IGV track of the 5mC CUT&Tag data in Extended Data Fig. 2b. These data highlight that libraries generated using the 5mC antibody on *in situ* nuclei produce very low signal with no discernible enrichment across genomic regions. Consistent with this, peak calling using MACS3 fails to identify any significant peaks.

This likely reflects a key technical limitation: antibody-based 5mC immunoprecipitation (e.g., MeDIP) typically requires denatured, single-stranded DNA, which is not achievable under *in situ* conditions in intact nuclei. Conversely, antibody-based CUT&Tag is not readily applicable to isolated DNA, as standard protocol steps such as washing are not feasible without immobilizing the DNA. These constraints further support the need for alternative approaches, such as our MBD–Tn5 fusion strategy, that can operate efficiently *in situ* and on purified DNA.

5) The DNA methylome of brain organoids has previously been described in Luo et al, Cell Rep 2026, and as such, the data presented in Figure 4 lack novelty. At the very least, the authors should benchmark the results obtained by MethCUT&Tag against this published data.

We have benchmarked our analysis throughout against Luo et al, Cell Rep 2016, and find high correlation between WGBS sequencing and C^{me}CUT&Tagenriched regions in iPSC/human ES cells (Fig. 1c-f, Extended Data Fig. 2b, f, g, h, j, Extended Data Fig. 3k, Extended Data Fig. 4a-c, Extended Data Fig. 6a). We have now expanded our previous comparison also towards their organoid data (Extended Data Fig. 7b-c). Despite using different developmental stages, we observe a similar trend, with dynamics of both lost and gained DNA methylation patterns in the organoids. We have also included loci showing dynamic methylation in https://www.biorxiv.org/content/10.1101/2025.10.01.679721v1.full, where the full data set is not yet available for more formal analysis.

6) Images should be presented of the cells and organoids that went into the genomics experiments.

Thank you for your suggestion. We have now included them in Extended Data Fig. 1c and Extended Data Fig. 7a.

7) The raw data was not accessible to the reviewer for evaluation.

We apologise for this and have now prepared everything on GEO with a reviewer link.

Reviewer #3 (Remarks to the Author):

This manuscript presents MethCUT&Tag, a promising new method that combines methylation-binding domain (MBD) fusion proteins with a hyperactive Tn5 transposase to target methylated regions of DNA for sequencing. The approach offers a potential powerful alternative for low-cost methylation profiling, and the data shown in human stem cells and organoid systems are convincing. The correlation with bisulfite sequencing is encouraging, and the demonstration of methylation dynamics during brain organoid development highlights the method's biological relevance. Overall, I am positive about the work and its potential impact, but there are several points that should be clarified or expanded to strengthen the manuscript.

The authors should briefly discuss how MethCUT&Tag fits within the landscape of current methylation-profiling technologies. In particular, single-molecule sequencing platforms such as PacBio and the more accessible Oxford Nanopore (ONT) now allow direct methylation detection and show strong correlation with WGBS. Mentioning these would provide a more balanced overview of existing approaches.

Thank you very much for your positive assessment of our work and for highlighting its potential impact. We appreciate your thoughtful suggestion to better contextualize C^{me}CUT&Tag within the broader landscape of DNA methylation profiling technologies.

We have now expanded the Discussion (lines 218–220) to provide a more balanced overview of current approaches, including single-molecule long-read sequencing platforms such as PacBio and Oxford Nanopore. In addition, we have generated a comprehensive comparative table positioning CmeCUT&Tag alongside other methylation-profiling technologies with respect to resolution, input requirements, cost, scalability, and technical complexity (Supplementary Table 3).

Since the method also relies on MBD binding, previous enrichment approaches such as MethylCap-seq (e.g., Brinkman et al.) should be cited. It would be helpful to clarify how MethCUT&Tag improves on these earlier method, whether in terms of resolution, input requirements, workflow simplicity, or the ability to work directly on chromatin.

Thanks a lot for your suggestion. We have now included comparisons to MeDIP, MethylCap-Seq, and MiGS in the comparative table (Supplementary Table 3) and expanded the discussion accordingly (Line 226-235). We have also benchmarked CmeCUT&Tag against these technologies where published data was available (Fig. 1d, Extended Data Fig. 4, Extended Data Fig. 8). We highlight that the main advantages of C^{me}CUT&Tag include reduced cost per sample, low input requirement (now shown in Extended Data Fig. 3a-b and f-g), simplification of the workflow, elimination of end repair and adapter ligation steps, and the ability to operate directly with nuclei in situ. This in situ compatibility further enables adaptation to droplet-based single cell technologies, as demonstrated in Fig. 2e-g.

The authors should provide an estimate of how many peaks are typically identified per sample and what fraction of the genome they cover. A simple genomic annotation breakdown, showing the proportions mapping to promoters, gene bodies, CpG islands, and enhancers, would make the data easier to interpret.

Thanks a lot for your comment. We agree that this was not highlighted well enough in the previous version and we have now dedicated more space to this in the text (line 75-83 and 123-131) and figures (Extended Data Fig. 2d and 3h). We have also performed additional analysis to annotate genomic regions beyond the ChromHMM annotation (Extended Data Fig. 2e and 4a).

Are there peaks that lack CpG dinucleotides altogether? If so, they may reflect sequence preferences or biases of the MBD domains rather than genuine methylation signals. Including a short analysis or control addressing this would strengthen confidence in specificity.

Thank you for this suggestion. To assess potential nonspecific binding by MBD2-Tn5, we analyzed the residual signal observed following DNA methylation inhibitor treatment. If this signal were driven by nonspecific transposition, it would not be expected to preferentially localize to CpG dense regions. Instead, the remaining signal is strongly enriched for CpG-rich loci, arguing against non-specific Tn5 activity (Extended Data Fig. 5c-d). We generally observe a high CG content in C^{me}CUT&Tag peaks (Extended Data Fig. 3j and 8d).

Consistent with this, global DNA methylation levels only decrease from ~80% to ~20% following inhibitor treatment, as reported previously (PMID: 34906184) and confirmed by our EM-Seq measurements (Extended

Data Fig. 5a-b). We therefore interpret the residual C^{me}CUT&Tag signal as originating primarily from genomic regions that retain DNA methylation despite pharmacological inhibition. This interpretation is further supported by the absence of signal in CpG islands classified as unmethylated (Extended Data Fig. 2g).

The manuscript should clearly state the recommended DNA or cell input amounts. If MethCUT&Tag performs well with very low input, this would be a notable advantage compared with WGBS or EM-seq and should be highlighted.

Thanks again for highlighting this point. We have now performed additional titration experiments to determine the optimal input amount requirements for C^{me}CUT&Tag (Extended Data Fig. 3a-b and f-g) and revised the corresponding sections of the manuscript (line 113-116 and 120-121). In addition, we have included Supplementary Table 3 to provide a comparative overview of input requirements across methylation-profiling technologies and all experiments performed in the manuscript (Supplementary Table 1).

Some discussion of the method's potential limitations would make the paper more balanced. WGBS, EM-seq, and long-read technologies are largely organism-agnostic, whereas MethCUT&Tag uses human MBD domains. It would be useful to clarify whether the technique has been tested or is expected to be compatible with mouse DNA, and whether it could be adapted for other species such as zebrafish, Xenopus and other non mammalian vertebrates. This would help readers understand how broadly the approach can be applied.

Thanks for your suggestion. We have now balanced the Discussion accordingly (line 231-234). In addition, this comment prompted us to directly test cross-species transferability, and we successfully generated DNA methylation profiles in zebrafish that closely match published MethylCap-Seq Data and WGBS datasets (Extended Data Fig. 8). Together, these results suggest that C^{me}CUT&Tag is transferable across species.

Point-by-Point: **A scalable Tn5-based method for genome-wide DNA methylation profiling in development and disease**

We sincerely thank all reviewers for their continued effort, careful evaluation, and highly constructive feedback throughout the revision process. Your insightful comments have been instrumental in strengthening the manuscript, both in terms of experimental rigor and clarity of presentation. In particular, your suggestions prompted us to perform additional experiments, refine our analyses, and more carefully position the scope and conclusions of our work. We believe that these revisions have significantly improved the overall quality, transparency, and impact of the study.

Reviewer #1 (Remarks to the Author):

The authors have satisfactorily addressed my previous concerns regarding specificity and benchmarking, and I appreciate the additional experiments and clarifications provided in the revised manuscript.

Thank you very much for your positive evaluation of our revisions and for recognizing the improvements in specificity and benchmarking.

The inclusion of a single-cell implementation of CmeCUT&Tag is interesting and represents a potentially important extension of the method. However, upon closer examination of the single-cell data, I have several concerns regarding the strength of the conclusions drawn from this single-cell analysis.

We would particularly like to thank you for your insightful suggestion during the revision process to further consider the method's single-cell applicability. This comment directly motivated us to expand our efforts beyond discussion and perform a dedicated single-cell experiment.

We found this direction to be highly valuable, as it yielded exciting results demonstrating that C^{me}CUT&Tag can be implemented in a droplet-based single-cell context. Importantly, this addition has substantially strengthened the manuscript by extending its scope toward the rapidly growing single-cell field and highlighting the future potential of the technology.

We further appreciate your detailed and thoughtful evaluation of the single-cell experiment. We agree that the current single-cell experiment primarily demonstrates compatibility with droplet-based workflows, rather than providing a comprehensive validation of the method’s ability to resolve subtle epigenetic differences at single-cell resolution, which is beyond the

scope of our manuscript. In response to your comments, we have carefully revised the manuscript to tone down our conclusions and now explicitly present the single-cell data as a proof-of-concept demonstration.

Furthermore, we have expanded the Methods section to provide additional details on cell input, recovery rates, and quality control metrics, to improve transparency and allow a more accurate assessment of the experiment.

We believe these revisions provide a more accurate and balanced interpretation of the single-cell results while clearly defining the current limitations and future potential of the approach.

Thank you again for these valuable comments, which have helped us substantially improve the clarity and positioning of this part of the manuscript.

First, the current experiment relies on a mixture of two highly distinct cell types (K562 leukemia cells and iPSCs), which are epigenetically very different. While the observed separation between these populations demonstrates compatibility with droplet-based workflows, it does not provide evidence that the method can resolve more subtle differences, such as closely related cell states or heterogeneous populations within tissues. As such, I would encourage the authors to temper their claims and restrict their conclusions to demonstrating compatibility with single-cell workflows.

Thank you for pointing this out. We have now adapted the text to:

Line 176-184: "Dimensionality reduction and unsupervised clustering revealed two main populations that broadly correspond to the two input cell types, with enrichment of K562 cells in one cluster and iPS cells in the other (Fig. 2g). While the separation is not complete and reflects the limited information content per cell, these results indicate that C^{me}CUT&Tag is compatible with droplet-based single-cell workflows and captures cell type–associated DNA methylation differences, albeit at a coarse level given the current data sparsity.

We note that this experiment is a proof of concept, and further optimization will be required to improve signal-to-noise ratios and potentially resolve more subtle differences between closely related cell states."

Second, the clustering results themselves appear relatively coarse. In Fig. 2g, both clusters contain a substantial fraction of the opposing cell type (i.e., K562 cells in cluster 0 and iPSCs in cluster 1), suggesting that separation is not clean but rather reflects a general trend. This raises concerns about signal sparsity, noise, or insufficient per-cell information content, and should be discussed more explicitly.

Thank you. We agree with the point you raised and have added it to the text.

Line 179-182 "While the separation is not complete and reflects the limited information content per cell, these results indicate that C^{me}CUT&Tag is compatible with droplet-based single-cell workflows and captures cell type–associated DNA methylation differences, albeit at a coarse level given the current data sparsity."

We have also now included the fragment number per cluster in the legend of Figure 2g, to document the signal sparsity (Line 1152-1152).

"(Each cluster contains an average number of 1058 and 1356 fragments that passed filters, respectively)."

Third, the overall recovery appears low. The experiment is described as starting from approximately 2 million cells, yet only ~1,100 cells are ultimately recovered, and ~500 cells per population are retained after filtering. It is unclear how many nuclei were loaded into the 10x Chromium controller and what the expected versus observed recovery rates were. Providing a detailed accounting of cell numbers at each step (input, loading, recovery, filtering) would greatly improve transparency and allow proper assessment of method efficiency.

Thank you for the suggestion. We have now adapted the methods section to include this information.

Line 493-494: "We obtained around 120 k nuclei at this point after the incubations, corresponding to around 15% of the initial input."

Line 497-501: "For the sample, 10 k nuclei were loaded onto the Chromium Next GEM Chip and processed following the manufacturer's protocol (steps 2–4: GEM generation and barcoding, post-GEM incubation cleanup, and library construction). After sequencing and before filtering, we obtained 1950 cells with valid barcodes from the experiment, corresponding to a recovery rate of 20% compared to what had been loaded."

Line 675-677: "After SNP demultiplexing, 1132 cells with an average of 1160 fragments per cell (median 754) were retained and used for downstream clustering analysis with FindNeighbors and FindClusters functions from Seurat[61] and Signac[62]."

Finally, key quality control metrics typically expected for single-cell chromatin assays are not clearly reported. For example, it is unclear whether FRiP scores, duplication rates, or other per-cell quality metrics were assessed and used for filtering. In addition, the number of biological or technical replicates for the single-cell experiment should be explicitly stated.

We have added this information to the methods section.

Line 667-672: "Single-cell C^{me}CUT&Tag data were mapped to hg38 using cellranger count, resulting in 1950 cells with valid 10x barcodes. After filtering for the number of counts per cell (>20), the number of features per cell (>20), nucleosome signal (<3.5), FRiP score (>15), and number of fragments passed filter (< 10000), 1799 cells were retained. The average number of fragments passed filtering per cell is 1054 (median 629), comparable to previous reports[10,60]; the average FRiP score is 29%."

Line 675-677: "After SNP demultiplexing, 1132 cells with an average of 1160 fragments per cell (median 754) were retained and used for downstream clustering analysis with FindNeighbors and FindClusters functions from Seurat[61] and Signac[62]."

We have also added the replicate number to the legend of Figure 2e (Line 1047-1048). "Cell lines were prepared independently and mixed during the nuclei isolation. One single-cell suspension was processed for the experiment".

Overall, while the single-cell extension is interesting, the current data should be presented more cautiously as a proof-of-concept demonstration of compatibility with droplet-based workflows, rather than as evidence of a mature or robust single-cell DNA methylation profiling platform. Clarification of the above points and a more balanced discussion would substantially strengthen this part of the manuscript.

Thank you again for your thoughtful and constructive feedback and for highlighting the importance of the single-cell extension. In response, we have carefully reframed this section as a proof-of-concept demonstration of compatibility with droplet-based workflows and have moderated our conclusions accordingly. We have further clarified the limitations of the current dataset, including the relatively coarse clustering and limited per-cell information content, and expanded the Methods and Figure descriptions to improve transparency regarding input material, recovery rates, and quality metrics. Together, these revisions provide a clearer and more rigorous presentation of the single-cell results.

Reviewer #1 (Remarks on code availability):

I did not run the code myself, but it appears to be well-structured and self-explanatory.

Reviewer #2 (Remarks to the Author):

In the revised manuscript, the authors provide several additional new data and analyses that significantly strengthen the work. Specifically, the authors show the applicability of the method by using it to classify different brain tumor types and by demonstrating its applicability to study zebrafish development. Moreover, the authors now provide more detailed benchmarking of the new CmeCUT&Tag method against existing approaches. The titration of input material further increases the applicability of the new method. The raw data is now also accessible via the GEO repository. In summary, my concerns have been satisfactorily addressed, and I recommend the manuscript for publication.

Thank you for your very positive and encouraging assessment. We greatly appreciate the recognition of the extensive revisions undertaken and your support for publication. Your feedback has been invaluable in helping us strengthen the manuscript. We are pleased that the inclusion of the tumor classification, zebrafish application, expanded benchmarking, titration experiments, and data availability has satisfactorily addressed your concerns.

We appreciate your recommendation for publication and thank you for your constructive feedback throughout the revision process.

Reviewer #3 (Remarks to the Author):

The authors have gone above and beyond in addressing the reviewers' comments. I congratulate them on their excellent work and fully support the publication of this manuscript.

Thank you for your very kind and encouraging feedback. We greatly appreciate your recognition of the revisions and your support for publication. Your comments have been invaluable in helping us improve the manuscript.
